# Participation in a school-based walking intervention changes the motivation to undertake physical activity in middle-school students

**DOI:** 10.1371/journal.pone.0204098

**Published:** 2018-09-25

**Authors:** Paolo Riccardo Brustio, Paolo Moisè, Danilo Marasso, Davide Alossa, Franco Miglio, Anna Mulasso, Emanuela Rabaglietti, Alberto Rainoldi, Gennaro Boccia

**Affiliations:** 1 NeuroMuscularFunction | Research Group, School of Exercise and Sport Sciences, Department of Medical Sciences, University of Turin, Turin, Italy; 2 School of Exercise & Sport Sciences, SUISM, University of Turin, Turin, Italy; 3 Istituto Comprensivo Statale Buttigliera Alta-Rosta, Scuola Secondaria di primo grado “G. Jaquerio”, Buttigliera Alta, Turin, Italy; 4 Istituto Comprensivo di Santena—Scuola Secondaria di primo grado "G. Falcone”, Santena, Turin, Italy; 5 Department of Psychology, University of Turin, Turin, Italy; University of Kentucky, UNITED STATES

## Abstract

The motivation to perform physical activity is a crucial factor in achieving a persistent active lifestyle. However, motivation decreases with increasing age from childhood to adolescence. The promotion of physical activity in educational settings might be an important tool to increase motivation and, consequently, to decrease sedentary behavior. The aim of this study was to explore the effect of a 4-month school-based walking intervention on motivation to participate in physical activity among Italian middle-school students. This study included 276 students (mean age 13 ± 1 years, 41.3% female). A total of 138 students (intervention group) participated in a brief walking intervention that was added to their routine daily school activity, while a convenience sample of 138 students performed the routine daily school activity. The activity consisted of walking one kilometer outside of school buildings during the morning break. Motivation data were collected before and after the walking breaks using the Participation Motivation Questionnaire (PMQ). Controlling for age, significant interactions between group and time were observed in the “Social Status” [F(1,273) = 4.851; *p* = 0.028], “Team” [F(1,273) = 6.015; *p* = 0.015] and “Energy Release” components [F(1,273) = 8.527; *p* = 0.038]. Specifically, a significant decrease in the “Social Status” components of the PMQ and an increase in the “Team” and “Energy Release” components were observed in the intervention group. On the contrary. control group showed an increase in “Social Status” and a decrease in the “Team” and “Energy Release” components. Within this developmental context, incorporation of the walking activity helped to modify the motivational orientation towards physical activity to include more intrinsic factors, which were related to the possibility of remaining with classmates and peer groups and releasing a surplus of energy.

## Introduction

Physical inactivity has become a global public health problem among children in developed countries [1], including Italy [2]. Indeed, physical inactivity and the consequent increase in sedentary behavior among children and adolescents induces several health problems, including overweight and obesity [3], as well as decreased cardiovascular fitness [4]. Although a minimum of 60 min per day of moderate-to-vigorous physical activity is suggested, few children (i.e., approximately 40%) follow this recommendation [3, 5–9]. Moreover, the physical activity level tends to decrease over time from childhood to adolescence [9, 10]. Thus, the increase of daily physical activity to the recommended levels is important for children’s health and well-being [7, 11].

Schools, particularly physical education classes, are a suitable place to promote all forms of physical activity [1, 12, 13]. Indeed, Italian physical activity action plans highlight the need to provide more opportunities to be active, particularly in childhood [14, 15]. For this reason, physical education programs are expected to promote an active and healthy lifestyle among children in schools [16]. However, the pressure due to grade testing, lack of time, and fear that the physical activity may negatively influence academic achievements are the most common barriers to physical activity in schools [17–20].

Motivation is an important factor that contributes to physical activity participation [21, 22]. It is a dynamic process that incorporates cognitive, affective, and values-related variables, which are assumed to mediate the choice and attainment of achievement goals [23, 24]. The Self-Determination Theory (SDT) [25] is a framework for examining the relation between motivation and physical activity [26]. In particular, the SDT posits that the emphasis on internal and external forces fits with the presence of two types of motivation, namely intrinsic and extrinsic motivation [27, 28]. Although intrinsic and extrinsic motivations are on a continuum, the first type may concern the pleasure and satisfaction derived from participation, while the latter may refer to the engagement of activity to obtain some types of reward, to gain in status or to avoid punishment [27–29].

Autonomous forms of motivation (i.e., internal forms) are positively related to change, support [29] and long-term maintenance of physical activity [21]. On the contrary, controlled forms of motivation (i.e., external forms) can be characterized by greater levels of instability and usually do not promote long-term positive physical activity behaviors [21]. In children, a persistent motivation to physical activity includes internal forces, such as skill development challenge, excitement, and fun, as well as external forces with a high grade of stability, such as the demonstration of competence and affiliation [28, 30].

Similarly to physical activity, motivation to physical activity decreases with increasing age from childhood to adolescence [31]. However, a recent study [32] has reported that children with higher level of intrinsic motivation and enjoyment, as well as a greater decline in extrinsic motivation (e.g., social and competence goals), demonstrated higher levels of physical activity. Specifically, a higher level of intrinsic motivation was positively associated with physical activity level during physical education classes and physical leisure time [26], as in non-educational context [8].

Brief active breaks during the school day are a relatively new and innovative method of increasing physical activity in educational settings [33–39]. These brief bouts of approximately 5–15 minutes can be considered to be an efficient and feasible intervention due to the short execution time and the relative low cost in a school context [33–35, 39]. Moreover, it does not require specific experience to be conducted in physical educational context.

In particular, brisk walking activities in school context might positively affect physical activity level and the general health of the children [40–43]. Due to the lower motor skills required compared to other activities and sports [44], walking can be performed by everyone, and it helps to reach the recommended levels of daily physical activity. Moreover, it requires that teachers have only a little specific experience in conducting physical education. For example, accumulated brisk walking in a school environment has been shown to increase daily energy expenditure [41], with a positive effect on the changes in body composition [42]. Indeed, brisk walking and running interventions in school may increase both light [43] and moderate to vigorous physical activities [40] and increase the fitness level in general.

To date, studies (for a review see [22, 45]) have shown that teaching methods and curriculum changes may improve students’ physical activity and motivation. For example, using a multi-component school-based intervention of 6 months, González-Cutre and colleagues [46] have observed a positive effect on students’ motivational variables and levels of physical activity. The interventions specifically comprised an extracurricular program of physical activity, meetings with families, and the inclusion of teaching units about fitness and health in the physical education classes [46]. Conversely, when the physical education intervention consisted only of additional vigorous activity, a negative impact on students’ motivation and future participation to physical activity was observed [12, 45]. Beyond this observation, to increase physical activity in a school context, it is necessary at the same time to create new opportunities for physical activity and to focus on enhancing intrinsic forms of motivation [32]. Despite these findings, to the best of our knowledge, no studies have investigated the effects of brief activity breaks on the motivation to participate in physical activity in children between 11 and 14 years of age. Thus, this study aimed to investigate whether participating in a program of active breaks might change the motivation to participate in physical activity in a sample of Italian middle-school students.

## Materials and methods

### Design and procedure

This study included two groups: an intervention and a control group. The intervention group participated in a brief walking intervention added to their routine daily school activity. A convenience sample (control group) was paired by gender and age and received the usual daily school activity. All participants were assessed before and after the four-month intervention period. The study was conducted for four months from February to May 2016.

Investigators obtained and recorded baseline assessments of motivation questionnaire. The same assessment was performed at the end of the intervention. The same trained and qualified investigators conducted the two assessment waves. Before testing, the following data were assessed for each participant: height, weight, and body mass index (BMI). A questionnaire was autonomously completed at school by each participant and in the presence of a researcher to clarify any questions. The questionnaire required approximately 10 minutes to complete.

### Participants

The sample included 276 students who were recruited from two middle schools in the neighborhood of Turin (Buttigliera Alta and Santena), in Italy. Specifically, students from one middle school followed the intervention, while a convenience sample (control group), paired by gender and age, was recruited from the other school. Both schools were part of the same institutional and regional school office (i.e., Ambito Territoriale di Torino), had similar facilities, identical educational curricula, and represented the standard Italian school. Therefore, any possible potential variances in education delivery impacting the outcome measurements were reduced [40]. All participants and their parents were informed that participation in the study was voluntary and confidential. The University of Turin Institutional Review Board approved the study. Before starting the study, the parents of each student read, agreed with and signed a written informed consent form, in accordance with Italian law and the ethical standards outlined in the 1964 Declaration of Helsinki.

Participants were assigned to the control or intervention group based on the middle school that they attended. The intervention group consisted of 138 students (mean age, 12.3 ± 0.9 years; 41.3% female), and similarly, the control group included 138 students (mean age, 12.9 ± 0.9 years; 41.3% female).

### Intervention of brief active breaks

The intervention program was inspired by a popular program named *“The daily mile”* [40, 47]. It consisted of walking for 1 km outside the school buildings along a path purposely marked in the schoolyard. The path was 350 m long, and students were required to walk it three times. All sessions started at 11:00 am, just before the recreation time, and lasted for approximately 10 minutes. The activity was conducted by the classroom teacher and did not require a specific training, as it simply consisted of walking. The intervention was programmed on a daily basis (from Monday to Friday) for four months. The only obstacle to executing the program was the rainfall (36% of the possible days), during which the teachers decided not to perform the activity. Consequently, the activity was performed 3 to 4 times a week over the four months of the intervention. No other event prevented participation in the activity.

### Motivation questionnaire

The Participation Motivation Questionnaire (PMQ) [48, 49] was used to assess the motivation to participate in physical activity. The PMQ is a self-reported questionnaire composed of 30 items investigating the possible reasons of students to participate in the physical activity. Originally, the questionnaire used a three-point scale and had an internal structure consisting of eight factors [48]. Later, Dwyer (1992) [49] developed a five-point scale for the ratings, with an internal structure of six factors and Cronbach [Alpha] coefficients ranging from 0.63 to 0.91 for the factors. In this study, for each item, a five-point Likert scale was used, ranging from 1 *Not at all important* to 5 *extremely important*. Higher scores indicated a higher motivation in physical activity.

In previous studies, the PMQ was adapted and used to investigate motivation to participate in physical activity in different contexts, such as physical education settings [50, 51], a sports science university [52], and older adults [53]. However, the factor analysis of these studies revealed a different number and composition of factors, suggesting that they were dependent on the sample under investigation. For this reason, as the PMQ presented a basic 6 to 8 factor structure, any use of a questionnaire requires the performance of a principal component analysis to identify these factors and the subsequent scale reliability [52, 54]. All students completed the questionnaire one week before beginning and one week after ending the intervention.

### Statistical analyses

A principal component analysis was performed to determine the factor structure of the PMQ, according to the study sample. According to Jones and colleagues [54], the items and factors were selected by considering the criteria of factor loadings above 0.40 and eigenvalues above 1.0. Internal consistency of the factors was examined using Cronbach’s Alpha. Values of α ≥ 0.70 were considered to be acceptable [55]. Then, a *t*-test analysis was performed for each PMQ component to assess baseline differences between the intervention and control groups. Thus, controlling for age, a series of one-way repeated measures ANOVAs with within factor times (i.e., baseline and post-test) and between-factor groups (i.e., intervention and control groups) were conducted to determine whether there were differences in the PMQ components. Differences between the treatment groups over time were determined by significant time × group interactions. The statistical significance level was set at *p* < 0.05. The Statistical Package for Social Sciences (SPSS 24.0 for Windows; IBM Corp, Armonk, NY, USA) was used for the analyses.

## Results

The sample presented a baseline mean BMI of 20.13 ± 3.93 kg m^-2^. Specifically, the BMI was 20.29 ± 3.95 kg m^-2^ for the intervention group and 19.96 ± 3.92 kg m^-2^ for the control group. No significant between-group differences were observed [t(273) = 0.700; p = 0.485].

Regarding PMQ, the overall Kaiser-Meyer-Olkin measure was 0.8, and Bartlett's test of sphericity was statistically significant (*p* < 0.001), indicating that the data could be likely decomposed into factors. Items and factors were selected by considering the criteria of factor loadings above 0.40 and eigenvalues above 1.0. The principal component analysis revealed eight factors (with eigenvalues higher than 1.0) that explained 22.39%, 10.67%, 7.44%, 6.07%, 5.21%, 4.07%, 3.81% and 3.53% of the total variance, respectively. The first component, named “Social Status”, was related to recognition and popularity (e.g., I want to be popular; I want to gain status or recognition); the second component, named “Team”, was related to cooperation and membership (e.g., I like the team spirit; I like being on a team); the third component, named “Competition”, was related to win and challenge (e.g., I like to compete; I like the challenge); the fourth component, named “Sport & Friend”, was related to the pleasure of being with friends and engaged in sport activity (e.g., I like to meet new friends; I like to get out of the house); the fifth component, named “Improve Skills”, was related to develop skills (e.g., I want to learn new skills; I want to improve my skills); the sixth component, named “Fitness & Health”, was related to physical wellbeing (e.g., I want to stay in shape; I want to be physically fit); the seventh component, named “Fun”, was related to the enjoyment for the action (e.g., I like the action; I like to have fun); the eighth component, named “Energy Release”, was related to canalize energy (e.g., I want to release tension; I want to release energy).

The total percentage of the explained variance (63.18%) was slightly higher than that previously reported by Kondric and colleagues [52] and Zahariadis and colleagues [56] (for more details, see Table 1). Because the fifth and seventh component (i.e., “Improve Skills” and “Fun”) did not present a sufficient level of internal consistency (Cronbach's alpha of 0.64 and 0.61, respectively), they were excluded from subsequent analysis.

**Table 1:** Factor structure of the PMQ (principal components, varimax rotation).

	Components							
Items	1	2	3	4	5	6	7	8
I want to gain status or recognition	**0.777**							
I want to be popular	**0.776**							
I like to feel important	**0.722**		0.377					
I like to do something I am good at	**0.470**				0.323			
I like the teamwork		**0.869**						
I like the team spirit		**0.868**						
I like being on a team		**0.853**						
I like to compete			**0.737**					
I like the challenge			**0.693**					
I like to win	0.396		**0.641**					
I like the rewards	0.310		**0.628**	0.336				
I like to meet new friends				**0.676**				
I want to be with my friends		0.378		**0.641**				
I like to travel				**0.611**				
I like to get out of the house				**0.556**				
My parents or friends want me to participate	0.454			**0.479**				
I want to learn new skills					**0.720**			
I want to improve my skills					**0.643**	0.354		
I like the excitement					**0.522**			
I want to reach a higher level	0.302		0.341		**0.516**			
I like the coaches or instructors			0.369	0.401	**0.486**			
I want to stay in shape						**0.837**		
I want to be physically fit						**0.830**		
I like to exercise						**0.554**	0.424	
I like to use the equipment or facilities							**0.648**	
I like the action							**0.627**	
I like to have something to do				0.377			**0.562**	
I like to have fun							**0.438**	
I want to release tension								**0.876**
I want to release energy								**0.844**
Cronbach’s alpha	0.772	0.893	0.820	0.717	0.645	0.740	0.616	0.752
Eigenvalues	6.717	3.201	2.231	1.821	1.563	1.221	1.142	1.058
Variance explained (%)	22.391	10.671	7.437	6.070	5.209	4.070	3.806	3.527

Notes: Component: 1 = Social Status; 2 = Team; 3 = Competition; 4 = Sports & Friends; 5 = Improve Skills; 6 = Fitness & Health; 7 = Fun; 8 = Energy Release

No baseline differences were observed between the two groups in “Team” [*t* (274) = −1,677, *p* = 0.095], “Fitness & Health” [*t* (274) = 0.498, *p* = 0.619] and “Energy Release” components [*t* (274) = 0.571, *p* = 0.568]. Conversely, at baseline, the control group reported higher values in “Social Status” [*t* (274) = −2.107, *p* = 0.036], “Competition” [*t* (274) = -2.014, *p* = 0.045] and “Sport & Friend” components [*t* (274) = -2.937, *p* = 0.004].

No significant difference was observed over the times (i.e., between baseline and post-test) in the overall PMQ score (i.e., sum of the different components) [from 75.51 to 75.28 points; F(1,274) = 0.124; *p* = 0.725]. Indeed, a similar trend was observed between the baseline and post-test in the intervention [from 73.47 to 73.43 points; F(1,137) = 0.002; *p* = 0.967] and control group [from 77.55 to 77.14 points; F(1,137) = 0.288; *p* = 0.592].

Table 2 shows the means and standard deviations of the control and intervention groups for the different components of the PMQ at baseline and post-test.

**Table 2:** Repeated measures analyses of variance of the PMQ components.

	Group				
	Intervention		Control		
Components	Baseline	Post-test	Baseline	Post-test	Time × Group
Social Status	13.76 ± 4.51	13.15 ± 4.23	14.89 ± 4.45	15.21 ± 4.46	**F = 4.851; *p* = 0.028**
Team	11.62 ± 3.68	11.77 ± 3.39	12.28 ± 2.78	11.72 ± 3.00	**F = 6.015; *p* = 0.015**
Competition	12.38 ± 4.34	11.92 ± 4.16	13.40 ± 4.08	13.57 ± 4.04	F = 3.220; *p* = 0.074
Sports & Friends	15.75 ± 4.56	16.44 ± 4.66	17.31 ± 4.23	17.18 ± 3.84	F = 2.980; *p* = 0.085
Fitness & Health	12.63 ± 2.44	12.43 ± 2.74	12.49 ± 2.39	12.37 ± 2.51	F = 0.394; *p* = 0.531
Energy Release	7.31 ± 2.19	7.69 ± 2.17	7.15 ± 2.22	7.07 ± 2.10	**F = 8.527; *p* = 0.038**

Notes: Data are presented as the mean and standard deviation (M ± SD).

**Fig 1:**
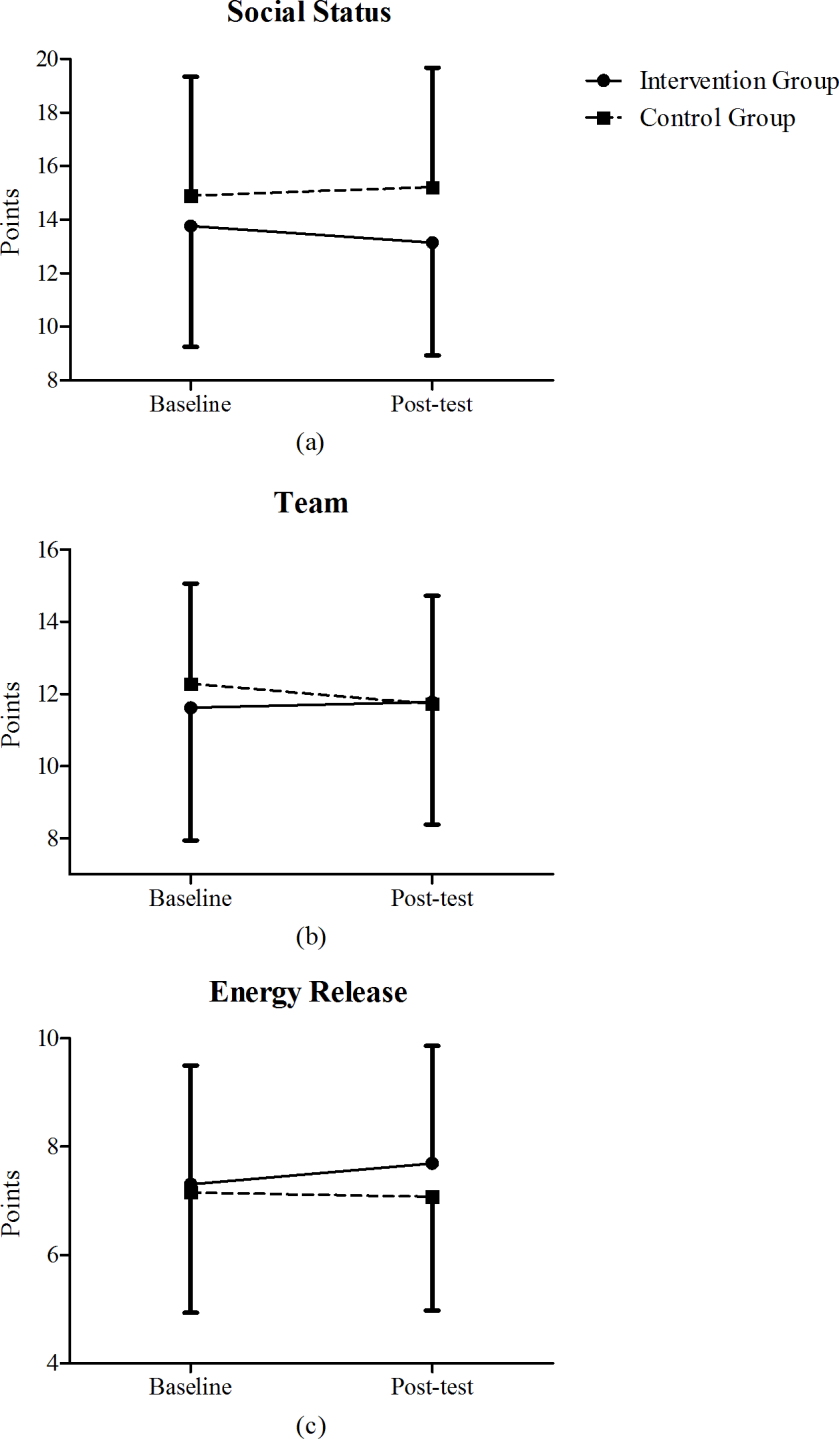
Social Status (a), Team (b) and Energy Release (c) components in the intervention (solid line) and control groups (dashed line) at baseline and post-test.

Controlling for age, significant time × group interactions between group and time were observed in the “Social Status” [F(1,273) = 4.851; partial η^2^ = 0.039; *p* = 0.028], “Team” [F(1,273) = 6.015; partial η^2^ = 0.022; *p* = 0.015] and “Energy Release” components [F(1,273) = 8.527; partial η^2^ = 0.038; *p* = 0.038]. Specifically, the “Social Status” component (Fig 1A) increased between baseline and post-test in the control group [from 14.89 to 15.21 points], whereas it decreased in the intervention group [from 13.76 to 13.15 points]. Moreover, a decrease in the “Team” component (Fig 1B) was observed between baseline and post-test in the control group [from 12.28 to 11.72 points], while the intervention group showed a small increase [from 11.62 to 11.77 points]. Finally, considering the “Energy Release” component (Fig 1C), a decrease was observed between baseline and post-test in the control group [from 7.15 to 7.07 points], while it increased in the intervention group [from 7.31 to 7.69 points].

Conversely, no significant time × group interactions were observed in the “Competition” [F(1,273) = 3.220; partial η^2^ = 0.012; *p* = 0.074], “Sports & Friends” [F(1,273) = 2.980; partial η^2^ = 0.011; *p* = 0.084] and “Fitness & Health” components [F(1,273) = 0.394; partial η^2^ = 0.001; *p* = 0.531] (For more details see Table 2).

## Discussion

This study aimed to assess whether participating in a walking-based break could change the motivation to participate physical activity among middle school students. Thus, we investigated the effect of participating in a 4-month program constituted by the inclusion of a 10-min walking-break during the school day. Specifically, the activity consisted of walking for 1 km along a path purposely marked in the schoolyard before the scheduled recreation time.

The intervention group showed an increase in the “Team” and “Energy Release” components (Fig 1), and a trend toward an increase was observed for “Sport & Friend” (Table 2). These changes in the shape of motivation may guide a child to practice physical activity for being included in a group (“Team”), or because he/she needs to canalize and release energy (“Energy Release”) or simply because he/she wants to have fun and enjoy the activity (“Sport & friend”). The increase in “Team” component is of particular interest in the school context. Indeed, schools tend to promote values such as teamwork, cooperation, and membership that are necessary of being part of social group [57]. An increase in the motivation to use physical activity as a socially acceptable way to canalize energy (“Energy Release” component) is another important improvement revealed by our data. Indeed, children spend most of their awake time in schools without the possibility to freely move and release tension. Using physical activity to canalize their energy in an healthy and constructive way may help to prevent antisocial behavior during the school day [58]. The observed decrease in “Social Status” (Fig 1), together with a non-significant trend toward a decrease in the “Competition” component (Table 2), were also salient findings of this study. Indeed, being involved in physical activity and sports only to be popular and to impress classmates, may have drawback effects in the school context. Conversely, a decrease in these motivational components may avoid unhealthy behaviors, such as taking risks and winning at all costs.

Taken together, these results showed that the intervention group changed the shape of motivation towards more *intrinsic* forces (i.e., autonomous forms of motivation) at the expense of *extrinsic* forces (i.e., controlled forms of motivation). According to SDT [25], this finding may be seen as an improvement in characteristics related to personal interests, values, and potentiality. It could be also related to an increase in feelings of enjoyment, pleasure, and satisfaction [25]. Furthermore, intrinsic motivation allows people to experience a positive and exciting context, which then provides opportunities to participate in challenging activities [27]. These results may be attributed to an increase in external interest, such as affiliation, which was characterized by high stability over time, but not by other external interests, such as fame, wealth, and appealing image which are less stable over time [28].

In general, our results suggest that the implementation of active breaks, based on a walking activity [44, 59], might have an effect on the motivation to physical activity among children and adolescents. Motivation plays a fundamental role in the promotion of physical activity [21, 46]. According to Pannekoek and colleagues [22], preserving motivation might play an important role in the development of adaptive physical activity habits that persist over childhood and adolescence. We speculate, as previously suggested [60], that students with an intrinsic motivation were more persistent in practicing physical activity at school and during leisure time, while extrinsic motivation did not show this type of relation. Indeed, even if our study did not measure physical activity level, we might speculate that the higher level of intrinsic motivation would be associated, over time, with higher levels of physical activity, as highlighted elsewhere [32]. These data may be particularly important to contrast the decreasing motivation [31] and physical activity level [9, 10] that are commonly observed over time through adolescence.

Despite the potential importance of these findings, several limitations should be noted. Our study should be replicated in a wider range of schools and ages to better generalize the results of our school-based interventions. The program was designed to minimize the time impact on the curriculum, and it simply composed of walking, thus, the intensity of the activity has not been investigated. Moreover, at baseline, the intervention and control groups differed in some motivational variables (i.e., “Social Status”, “Competition” and “Sport & Friend”). Since we could not randomize the classes participating in the program, differences at baseline may be expected. However, these differences would make any effects of the intervention harder to observe, not easier [40]. Notwithstanding, significant time × group interactions were observed despite these differences. Furthermore, we focused on the changes over time and observed a different pattern in the PMQ components between the groups. Indeed, the changes observed in PMQ components were more pronounced in the intervention group than in the control group. Another limitation could be related to the seasonal variation that might have influenced participants’ motivations. For example, environmental factors, such as weather or daylight hours, might impact the participants’ motivations. Future studies should investigate these aspects. Moreover, no data about physical activity levels were collected. It may be possible that students compensated for this increase in walking by reducing their physical activity in other points of the school day. Again, the study lacked physical health-related measures, such as cardiorespiratory fitness and muscular strength, as well as any variations in the BMI that may have occurred during the program; these aspects could be included in subsequent enlargement and in-depth studies. Thus, future research is necessary to better investigate changes in physical activity level and the long-term effect of this walking activity on physical outcomes. Finally, the intervention and control groups were recruited from two different schools, which may be a limitation due to the different contexts. However, the schools were part of the same school district, with the same delivery of education.

## Conclusion

In conclusion, our results showed that performing a brief walking break during the routine school day changed the motivation to perform physical activity. Specifically, incorporating a walking activity helped to direct the motivation to physical activity towards more intrinsic factors, which were related to the possibility of staying with classmates and peer groups and releasing a surplus of energy. These changes could be beneficial for the school context because they bring positive attitudes that fit with school prerogatives. We believe that our findings may encourage the inclusion of a brief active break, based on a walking activity, into the school day routine.

## Supporting information

S1 Dataset Data from participants in this study. (PDF)
