# Supplementary material for: Participation in a school-based walking intervention changes the motivation to undertake physical activity in middle-school students
Source: PLoS One. 2018 Sep 25;13(9):e0204098. doi: 10.1371/journal.pone.0204098 (PMC6155517; doi:10.1371/journal.pone.0204098)
Supplement: S1 Dataset — (PDF) [file pone.0204098.s001.pdf]

Group	Gender	Class	Age	T0_MOT_1_1	T0_MOT_2_1	T0_MOT_3_1	T0_MOT_4_1
0,00	1	1	11,8600	5	5	3	4
0,00	2	1	11,8500	4	4	1	3
0,00	2	1	11,8200	5	5	3	2
0,00	1	1	11,7700	5	3	5	4
0,00	1	1	11,7100	5	5	4	5
0,00	2	1	11,6700	3	5	1	4
0,00	2	1	11,5900	5	5	1	4
0,00	2	1	11,4900	3	3	2	3
0,00	1	1	11,4800	5	5	2	5
0,00	1	1	11,4700	5	4	1	5
0,00	1	1	11,4500	5	5	4	4
0,00	1	1	11,4100	5	5	2	4
0,00	2	1	11,3400	2	5	2	5
0,00	2	1	11,2200	5	5	4	5
0,00	1	1	11,1600	4	5	3	4
0,00	2	1	11,1500	3	3	3	5
0,00	1	1	11,0200	4	5	3	3
0,00	1	1	11,0100	3	4	3	3
0,00	1	1	10,9800	5	5	2	5
0,00	2	1	10,9500	5	3	2	3
0,00	2	1	10,9200	4	3	2	5
0,00	1	1	10,9200	5	5	2	5
0,00	1	1	10,9200	5	3	4	4
0,00	1	1	10,9000	4	5	3	4
0,00	2	1	10,8700	5	3	2	2
0,00	2	1	12,5700	5	5	3	3
0,00	1	1	11,7900	4	2	3	3
0,00	1	1	11,7800	5	4	3	1
0,00	2	1	11,7500	4	4	2	1
0,00	2	1	11,7300	2	3	1	5
0,00	1	1	11,6500	3	5	2	5
0,00	1	1	11,6300	4	5	2	3
0,00	1	1	11,5000	5	4	3	5
0,00	1	1	11,4900	5	4	3	3
0,00	2	1	11,4700	5	4	1	3
0,00	2	1	11,4400	4	2	1	3
0,00	1	1	11,3500	5	5	3	4
0,00	2	1	11,2900	4	5	2	5
0,00	2	1	11,2300	5	2	4	4
0,00	2	1	11,1500	4	2	4	3
0,00	1	1	11,1200	5	3	2	3
0,00	1	1	11,0400	5	4	3	5
0,00	2	1	11,0400	5	3	3	2
0,00	1	1	11,0300	5	4	3	5
0,00	1	1	11,0200	5	5	3	4
0,00	2	1	11,0100	5	3	4	5
0,00	1	1	10,9300	5	5	4	3
0,00	1	1	10,9100	5	5	3	4
0,00	1	1	10,8700	5	2	3	5

0,00	2	1	10,7700	5	3	2	2
0,00	1	2	12,7300	3	5	5	1
0,00	1	2	12,6700	4	5	2	4
0,00	1	2	12,6600	5	5	4	4
0,00	1	2	12,6100	5	3	4	5
0,00	2	2	12,5300	3	4	1	5
0,00	1	2	12,5100	2	3	4	5
0,00	1	2	12,5000	4	5	3	5
0,00	1	2	12,4400	5	5	4	5
0,00	1	2	12,4100	5	5	4	3
0,00	1	2	12,3200	4	1	2	5
0,00	2	2	12,2300	5	2	1	4
0,00	1	2	12,1700	5	1	4	3
0,00	1	2	12,1400	4	4	3	3
0,00	1	2	12,1200	4	2	3	3
0,00	2	2	12,0900	3	4	5	4
0,00	2	2	11,9900	3	5	2	3
0,00	2	2	11,9900	2	5	1	1
0,00	2	2	11,9800	5	2	4	1
0,00	1	2	11,9300	5	4	1	3
0,00	1	2	11,9200	3	1	4	3
0,00	1	2	11,8900	5	5	5	5
0,00	1	2	11,8400	5	5	5	5
0,00	2	2	11,7700	5	5	4	4
0,00	1	2	12,9000	5	4	5	5
0,00	2	2	12,8700	4	1	1	5
0,00	1	2	12,8000	5	3	4	5
0,00	1	2	12,7800	5	5	4	3
0,00	1	2	12,7700	5	4	4	4
0,00	1	2	12,5200	5	2	3	5
0,00	2	2	12,5100	5	4	3	1
0,00	1	2	12,5000	5	1	4	3
0,00	2	2	12,4800	4	3	3	4
0,00	1	2	12,4100	5	3	4	5
0,00	1	2	12,3800	4	2	5	3
0,00	1	2	12,3400	5	3	3	4
0,00	1	2	12,3400	5	5	5	5
0,00	1	2	12,3200	4	3	2	1
0,00	1	2	12,3100	5	2	1	4
0,00	2	2	12,2400	5	5	2	2
0,00	1	2	12,1600	5	4	2	5
0,00	1	2	12,0500	5	4	4	4
0,00	1	2	12,0400	5	2	5	1
0,00	1	2	12,0000	5	3	4	5
0,00	2	2	11,9000	4	3	2	2
0,00	2	2	11,9000	4	5	4	4
0,00	1	3	14,2800	4	2	1	1
0,00	2	3	13,7500	5	4	4	3
0,00	1	3	13,7200	5	1	4	4
0,00	1	3	13,7200	4	2	1	2

0,00	2	3	13,6900	5	1	2	3
0,00	2	3	13,6500	5	4	3	4
0,00	1	3	13,6000	4	3	4	2
0,00	2	3	13,5900	4	2	1	1
0,00	2	3	13,5700	4	2	1	4
0,00	1	3	13,5600	3	2	5	4
0,00	2	3	13,5100	3	2	4	5
0,00	1	3	13,4800	3	1	5	4
0,00	1	3	13,4600	4	2	5	5
0,00	1	3	13,3500	4	1	1	1
0,00	1	3	13,3300	3	4	5	2
0,00	2	3	13,3100	5	3	2	3
0,00	1	3	13,3000	5	2	2	5
0,00	1	3	13,2800	5	4	5	3
0,00	2	3	13,2300	4	2	3	5
0,00	2	3	13,2300	5	4	1	3
0,00	1	3	13,0500	4	4	2	4
0,00	2	3	13,0000	4	4	5	4
0,00	2	3	13,8700	3	5	4	3
0,00	2	3	13,8000	3	4	2	4
0,00	1	3	13,6900	4	4	1	4
0,00	2	3	13,6600	5	3	2	2
0,00	2	3	13,6300	5	2	3	4
0,00	2	3	13,5900	5	2	3	4
0,00	2	3	13,5100	3	3	3	2
0,00	1	3	13,4800	3	5	3	2
0,00	1	3	13,4700	5	4	5	3
0,00	2	3	13,4100	2	4	2	3
0,00	2	3	13,3400	5	4	5	5
0,00	1	3	13,2800	5	5	5	3
0,00	1	3	13,2200	4	5	5	5
0,00	1	3	13,2100	3	3	4	4
0,00	1	3	13,2000	5	4	3	4
0,00	2	3	13,1400	4	2	2	3
0,00	2	3	13,1100	5	2	5	5
0,00	1	3	13,1000	4	4	3	3
0,00	1	3	13,0900	4	3	2	3
0,00	2	3	13,0800	4	3	1	5
0,00	2	3	12,9600	5	3	4	2
1,00	1	1	14,5100	4	5	2	3
1,00	1	1	12,3800	4	3	2	3
1,00	2	1	12,2500	5	4	5	2
1,00	1	1	12,2000	4	5	2	4
1,00	2	1	12,1300	3	5	4	3
1,00	2	1	11,9900	4	5	3	4
1,00	1	1	11,9100	4	4	5	3
1,00	1	1	11,8900	5	3	2	2
1,00	2	1	11,8600	5	3	2	4
1,00	1	1	11,8300	3	3	5	5
1,00	2	1	11,7900	4	3	5	3

1,00	2	1	11,7900	5	4	4	1
1,00	1	1	11,7500	5	5	3	5
1,00	1	1	11,6300	5	2	5	1
1,00	2	1	11,5600	5	3	2	4
1,00	1	1	11,5600	4	5	4	4
1,00	2	1	11,5300	5	3	2	1
1,00	1	1	12,4000	5	5	4	5
1,00	2	1	12,0800	3	5	2	5
1,00	2	1	12,0800	5	5	5	5
1,00	2	1	12,0400	5	2	2	5
1,00	2	1	11,9300	5	5	1	4
1,00	2	1	11,9200	5	5	3	5
1,00	2	1	11,9100	4	3	3	1
1,00	1	1	11,8300	4	5	5	4
1,00	1	1	11,8200	5	3	1	5
1,00	2	1	11,7400	5	5	3	5
1,00	2	1	11,7200	3	5	3	4
1,00	1	1	11,6200	4	5	1	1
1,00	2	1	11,5000	4	5	3	3
1,00	1	1	11,4800	3	3	2	2
1,00	2	1	11,4000	4	5	2	1
1,00	1	1	12,4400	5	3	2	4
1,00	2	1	12,2300	4	5	3	4
1,00	2	1	12,1600	4	5	2	3
1,00	1	1	12,0600	5	5	3	4
1,00	1	1	12,0600	3	3	3	4
1,00	2	1	12,0400	5	4	4	3
1,00	2	1	12,0100	4	5	4	5
1,00	2	1	11,9900	3	5	2	2
1,00	1	1	11,8800	4	3	3	2
1,00	1	1	11,8500	4	5	5	4
1,00	1	1	11,7500	3	2	1	5
1,00	2	1	11,7400	4	2	2	5
1,00	2	1	11,6300	4	5	1	5
1,00	2	1	11,4600	3	3	1	2
1,00	1	1	11,9700	5	5	5	3
1,00	1	1	11,8700	5	5	4	5
1,00	1	1	11,5100	5	5	5	5
1,00	1	1	11,5100	5	5	5	3
1,00	1	2	13,3900	4	4	5	3
1,00	1	2	13,3500	5	5	3	4
1,00	1	2	13,2700	4	4	4	4
1,00	1	2	13,2100	5	5	3	4
1,00	1	2	13,1700	5	4	3	4
1,00	1	2	13,1100	4	5	3	4
1,00	1	2	13,0600	5	4	2	3
1,00	1	2	13,0100	5	4	2	3
1,00	2	2	13,4200	5	5	3	3
1,00	1	2	13,3900	5	5	5	4
1,00	1	2	13,3500	5	5	1	3

1,00	1	2	13,3500	5	4	2	3
1,00	1	2	13,3300	4	5	3	4
1,00	2	2	13,2900	4	2	4	5
1,00	2	2	13,2400	5	4	4	2
1,00	2	2	13,1900	5	5	4	4
1,00	1	2	13,0000	5	3	1	5
1,00	2	2	12,9600	5	5	3	4
1,00	2	2	12,9500	5	2	1	2
1,00	1	2	12,8100	5	5	5	4
1,00	1	2	12,7200	5	5	4	4
1,00	1	2	12,7000	5	5	3	1
1,00	2	2	12,6400	4	3	3	4
1,00	2	2	12,6000	5	4	5	4
1,00	1	2	12,4900	5	5	5	4
1,00	1	2	13,4300	5	4	5	5
1,00	2	2	13,2300	5	5	2	1
1,00	1	2	13,2100	3	5	4	3
1,00	1	2	13,1700	4	5	5	5
1,00	2	2	13,0600	5	5	5	2
1,00	1	2	12,9700	5	3	5	4
1,00	2	2	12,9100	4	3	4	2
1,00	2	2	12,8900	4	5	5	3
1,00	2	2	12,7600	5	4	4	5
1,00	1	2	12,7200	3	3	2	3
1,00	2	2	12,6800	5	5	5	5
1,00	2	2	12,6700	5	4	5	3
1,00	1	2	12,6400	5	4	4	5
1,00	2	2	12,6000	5	3	2	1
1,00	2	2	12,5800	5	3	1	1
1,00	1	2	13,2700	5	3	5	4
1,00	1	2	13,2300	3	3	3	2
1,00	1	2	13,2100	4	5	4	4
1,00	1	2	13,0100	5	3	5	4
1,00	1	2	12,5900	5	5	5	5
1,00	1	3	14,9700	4	3	4	4
1,00	2	3	14,4000	5	2	5	5
1,00	2	3	14,3500	4	3	3	5
1,00	2	3	14,2400	4	3	3	5
1,00	2	3	14,1300	5	4	2	3
1,00	1	3	14,0700	5	3	4	5
1,00	1	3	14,0500	4	5	5	5
1,00	2	3	14,0100	2	4	3	4
1,00	1	3	13,9600	5	5	3	4
1,00	2	3	13,8400	4	3	1	4
1,00	2	3	13,7100	3	2	5	4
1,00	1	3	13,6600	5	4	2	3
1,00	2	3	13,5500	5	3	1	5
1,00	1	3	13,5000	3	1	1	3
1,00	1	3	13,4600	5	3	4	1
1,00	1	3	14,1900	4	5	3	4

1,00	1	3	14,0300	5	3	2	5
1,00	1	3	14,0000	3	2	2	1
1,00	2	3	13,8700	4	1	2	5
1,00	2	3	13,8600	4	5	3	5
1,00	2	3	13,8300	5	4	3	3
1,00	1	3	13,8200	4	5	4	4
1,00	1	3	13,7900	5	1	1	5
1,00	2	3	13,7500	5	3	2	4
1,00	1	3	13,7200	5	4	4	3
1,00	2	3	13,6700	3	4	3	4
1,00	1	3	13,6300	3	5	3	5
1,00	2	3	13,6000	4	4	2	4
1,00	1	3	13,9500	3	1	5	5
1,00	1	3	13,7900	5	4	5	4
1,00	1	3	13,7700	5	5	5	4
1,00	1	3	13,6700	5	3	3	4
1,00	1	3	13,6600	5	5	5	5
1,00	1	3	13,5300	5	5	4	4
1,00	1	3	13,4500	4	4	3	4
1,00	1	3	14,4100	5	4	5	5
1,00	1	3	14,3800	5	3	4	5
1,00	1	3	14,2800	3	4	5	1
1,00	1	3	14,1200	3	5	4	2
1,00	1	3	14,0800	3	5	4	3
1,00	1	3	14,0600	4	1	1	4
1,00	1	3	13,7300	3	2	2	5
1,00	1	3	13,4600	2	1	1	3

T0_MOT_5_1	T0_MOT_6_1	T0_MOT_7_1	T0_MOT_8_1	T0_MOT_9_1	T0_MOT_10_1	T0_MOT_11_1
4	5	5	4	5	5	3
5	2	4	5	1	4	5
2	5	5	3	1	5	5
3	5	5	1	2	1	3
5	5	5	5	5	5	5
5	2	5	5	4	5	5
5	5	5	5	5	5	5
1	5	5	5	5	4	4
4	5	5	5	1	5	5
3	5	3	5	3	5	4
3	5	3	3	5	4	5
5	4	3	1	1	4	5
4	5	5	5	1	4	5
4	4	5	5	5	4	5
2	5	4	4	3	2	5
3	2	5	5	1	3	3
3	4	3	5	1	4	4
2	4	4	4	2	3	4
3	5	5	5	1	4	5
3	4	5	5	2	5	2
3	3	5	5	5	4	5
5	5	5	5	5	5	5
4	4	4	3	4	4	4
2	4	4	5	5	4	4
4	5	5	5	4	5	4
2	4	3	4	4	5	4
5	4	3	2	3	3	2
1	2	3	5	1	5	3
1	3	5	5	1	2	2
1	2	5	5	1	3	4
4	5	5	4	1	5	5
3	5	4	5	2	4	5
3	4	4	5	2	3	4
3	5	5	5	1	5	4
2	4	4	4	1	5	4
1	4	5	4	2	4	2
3	3	4	5	2	5	5
4	5	4	5	1	3	4
2	3	4	4	1	5	2
2	5	4	3	1	3	2
5	5	5	4	1	3	4
2	4	5	5	4	5	5
3	3	3	2	3	4	3
3	5	3	5	4	5	5
4	5	5	5	4	5	5
1	5	5	5	1	5	4
5	5	5	4	3	5	5
5	5	5	5	4	5	5
5	5	5	4	1	5	2

3	5	2	2	1	5	3
5	5	5	5	2	4	3
2	5	5	3	1	4	4
5	5	4	3	3	5	3
5	5	3	5	4	5	3
1	1	5	1	1	2	1
4	5	4	4	2	5	1
2	5	5	5	1	4	5
3	5	5	5	1	5	4
2	4	5	4	1	5	4
1	3	5	1	1	5	2
3	5	5	5	1	4	3
2	5	5	3	1	5	1
3	3	3	3	2	3	3
2	5	3	3	1	5	4
3	4	5	5	1	3	4
4	5	5	5	5	5	4
1	4	1	3	1	4	1
2	4	3	2	1	5	3
5	5	3	4	1	3	3
3	5	2	1	2	4	1
5	5	5	5	1	5	4
5	5	5	4	5	5	5
4	5	5	4	1	5	4
5	5	5	5	4	5	4
4	5	4	4	2	3	2
5	5	5	4	2	5	4
5	5	4	5	4	5	5
2	4	4	5	1	4	3
2	5	5	5	1	4	2
5	5	4	5	3	4	5
5	4	5	5	1	5	2
2	4	4	5	1	4	2
2	5	5	1	1	5	3
2	5	5	4	1	4	2
4	5	5	4	1	5	5
1	5	5	3	1	5	5
1	5	5	1	1	4	3
2	5	4	3	1	5	2
2	5	5	4	1	5	4
4	5	4	3	2	5	5
5	4	5	5	2	5	4
5	5	5	5	4	4	4
2	4	5	2	5	5	5
2	1	3	4	2	5	4
5	5	4	5	3	4	5
2	4	3	3	2	4	3
4	4	5	5	1	5	3
1	4	5	2	1	5	2
2	3	5	4	1	2	2

2	4	3	3	1	5	1
3	5	5	1	1	5	4
3	5	4	5	1	3	4
1	4	4	2	1	3	2
3	4	5	2	1	5	4
5	5	4	2	4	3	4
4	4	5	1	1	4	2
1	4	5	4	1	4	1
2	5	4	4	1	4	3
1	5	2	5	1	4	3
3	3	3	2	2	3	4
1	5	4	2	5	5	3
1	5	5	1	1	5	3
1	4	5	5	1	4	4
2	4	5	3	2	3	2
3	4	5	5	1	5	5
1	4	3	3	1	3	2
1	3	3	3	1	4	4
5	5	5	3	2	5	4
5	4	2	4	3	4	3
2	4	3	5	1	3	3
2	3	4	5	1	5	3
1	4	5	5	5	4	4
1	5	5	5	4	4	3
4	5	2	4	4	5	5
2	2	4	3	2	3	5
4	5	3	4	2	5	5
2	5	3	2	2	2	4
4	5	5	2	5	5	4
1	5	5	3	1	5	3
3	5	5	4	5	5	5
2	4	3	4	1	4	3
4	5	5	5	3	5	5
3	5	4	3	1	4	4
4	2	4	2	2	4	2
5	3	3	1	3	3	4
4	3	4	5	1	4	3
1	5	2	1	1	3	4
1	3	3	2	1	5	3
4	4	5	5	5	5	5
5	3	3	5	4	3	5
1	5	5	2	1	4	3
5	4	4	5	5	5	5
2	4	1	3	1	3	4
2	4	4	5	2	3	4
4	3	4	4	4	4	3
3	3	2	4	1	5	3
1	5	5	5	2	5	5
5	5	4	4	3	4	5
4	5	2	2	3	5	5

4	3	5	5	1	4	3
5	5	4	5	5	5	2
3	5	5	4	5	4	3
1	3	4	5	1	2	4
5	5	5	5	5	5	5
2	3	5	4	1	4	5
4	5	5	5	1	5	5
5	2	3	4	5	2	3
2	5	4	5	1	5	4
2	5	4	4	3	5	2
2	5	3	4	2	3	4
3	5	5	5	3	5	5
2	3	4	4	1	4	2
5	3	2	1	5	3	2
1	4	5	4	3	5	3
5	5	4	5	4	4	4
5	3	2	4	4	3	4
4	3	5	3	1	5	4
3	4	3	5	1	3	4
3	1	5	2	1	3	3
4	3	5	5	3	5	5
5	5	5	4	4	5	5
3	4	2	5	4	4	5
1	4	4	5	3	2	2
2	4	3	4	1	4	3
5	4	3	3	4	5	4
1	4	4	5	1	4	2
5	2	3	4	5	3	4
4	5	3	4	3	5	4
3	4	3	4	1	5	3
4	5	5	4	2	4	3
3	5	4	3	1	5	3
1	4	5	5	1	5	2
5	2	4	5	4	5	5
2	3	4	4	1	5	2
4	5	5	2	5	5	4
1	5	5	5	5	5	5
4	5	5	5	4	5	5
3	5	4	1	5	5	5
4	5	4	5	4	5	4
1	5	5	5	1	3	5
5	5	4	5	5	5	5
4	5	5	5	2	5	4
2	5	4	5	2	4	5
4	3	3	4	2	3	4
4	5	4	5	1	4	4
5	3	3	5	4	5	3
4	5	3	4	3	5	4
5	4	5	4	3	5	5
4	5	5	2	3	5	5

5	5	3	5	3	5	5
4	4	4	5	5	4	3
1	4	5	4	1	4	1
3	4	5	5	4	5	3
5	5	4	5	2	4	3
1	1	5	3	1	5	2
5	5	4	4	1	5	5
2	5	5	4	2	5	2
5	5	5	5	1	3	4
5	5	5	5	1	3	4
4	3	5	5	4	5	5
5	5	5	4	1	4	4
5	5	3	4	4	5	3
5	5	5	5	1	3	4
3	4	3	5	5	4	4
5	4	5	5	3	4	4
2	2	4	3	4	5	2
3	3	4	5	5	4	3
4	4	5	5	4	3	3
3	4	3	4	1	4	2
3	4	2	4	3	3	2
2	5	5	4	1	3	5
4	3	3	2	3	5	5
5	4	3	3	2	3	3
3	5	4	3	5	4	3
4	4	5	5	2	4	5
3	5	2	2	5	4	4
1	1	4	5	4	5	4
1	4	2	2	1	2	1
5	4	5	5	3	5	5
3	3	3	4	2	3	2
4	4	5	5	5	5	5
3	5	4	5	1	5	4
5	5	5	5	4	5	5
3	5	3	4	3	4	4
3	5	5	5	1	3	3
5	5	3	4	5	4	1
4	4	5	3	2	4	4
5	4	4	4	1	3	3
3	5	5	5	4	5	3
4	4	2	5	3	4	3
3	3	5	4	1	3	3
5	5	2	5	4	4	5
4	5	4	5	2	4	4
5	5	4	1	1	4	2
2	5	5	4	1	4	4
1	4	5	3	2	5	3
3	4	5	3	5	3	3
3	5	4	3	1	5	5
2	5	4	4	1	4	5

5	4	5	4	4	5	4
4	5	4	1	3	2	2
5	5	3	1	1	5	1
5	5	4	5	2	4	5
4	5	5	5	1	4	4
4	4	3	4	1	5	5
1	5	1	1	1	1	1
4	5	5	5	4	5	4
4	4	5	3	2	4	3
5	4	2	4	1	2	4
5	4	5	4	3	5	4
4	4	2	3	1	3	4
3	2	4	2	1	4	3
2	4	3	2	3	4	3
5	5	4	4	4	5	4
2	5	3	4	4	1	1
5	5	5	5	5	5	2
4	5	5	5	3	5	5
5	5	5	3	3	5	5
5	4	3	3	1	4	5
3	4	5	5	1	4	3
3	4	4	2	5	5	4
4	3	4	1	1	5	2
2	4	1	4	2	3	5
4	5	5	3	2	4	3
1	5	5	4	1	2	3
1	2	4	5	1	1	3

T0_MOT_12_1	T0_MOT_13_1	T0_MOT_14_1	T0_MOT_15_1	T0_MOT_16_1	T0_MOT_17_1
5	3	1	5	2	5
5	3	2	4	5	4
5	4	3	5	5	2
5	5	2	5	1	5
5	5	3	5	5	5
4	4	2	2	5	4
4	5	2	5	5	4
3	4	1	3	2	4
4	4	2	5	5	5
5	5	1	5	5	5
5	4	4	5	4	5
3	4	1	4	5	4
5	4	5	5	5	5
4	4	4	4	5	5
5	5	4	3	5	4
4	4	3	2	4	5
4	3	2	4	4	4
4	5	2	4	3	5
4	2	2	5	5	5
3	2	4	4	5	3
5	5	2	2	5	5
4	5	5	4	5	5
4	4	4	4	4	4
3	5	4	3	3	4
5	3	5	5	4	2
5	4	4	5	5	5
5	3	3	2	3	4
4	2	1	4	1	3
2	1	2	3	3	2
4	5	1	3	4	5
3	5	3	4	5	5
2	4	1	3	4	4
5	5	2	5	4	5
5	5	2	5	3	4
5	3	1	4	2	2
3	4	1	5	3	4
4	2	2	5	3	5
3	2	3	4	5	4
5	3	3	4	2	3
2	3	3	4	1	2
5	5	1	3	2	1
4	5	2	4	5	4
4	3	2	3	2	2
5	5	5	4	5	5
5	5	2	5	5	5
3	4	1	5	4	5
5	3	4	5	5	5
4	4	3	5	5	5
5	5	4	5	5	5

3	2	2	4	3	2
4	2	5	4	3	5
2	3	2	3	2	4
5	5	4	5	5	5
5	5	3	3	5	4
2	5	1	1	3	1
5	5	3	4	5	4
4	5	4	4	4	4
3	4	4	5	4	5
5	3	2	4	4	4
2	5	1	3	2	5
4	5	1	5	4	5
5	2	2	5	2	5
3	5	2	3	4	4
4	4	4	5	5	4
5	4	1	3	1	4
5	4	2	5	5	5
3	3	2	2	1	1
5	2	1	3	1	1
4	3	1	5	3	5
3	3	4	4	4	3
4	5	5	4	3	4
5	1	5	5	5	5
5	4	3	5	4	5
4	4	5	4	3	3
5	5	3	4	4	4
5	5	4	4	4	5
5	3	2	4	4	5
4	4	3	4	4	4
5	5	1	4	3	5
4	1	3	5	4	2
5	3	4	5	3	5
4	4	2	5	2	3
5	5	2	5	2	5
1	5	4	3	1	5
5	4	3	4	5	4
5	5	4	4	4	4
4	1	2	5	3	3
3	2	1	3	1	2
5	5	2	5	5	4
5	4	2	5	5	5
4	3	3	4	4	4
5	1	5	5	5	5
5	4	4	4	4	5
5	2	2	3	1	2
5	3	5	5	5	5
3	2	1	3	3	3
4	2	1	4	4	5
4	4	3	5	2	5
5	2	1	4	4	3

3	2	2	5	2	4
5	5	2	5	3	1
5	2	3	4	5	3
1	1	1	1	4	1
2	3	1	3	2	3
5	5	4	5	3	3
5	5	4	4	4	1
3	4	4	5	3	4
5	4	5	4	4	4
4	2	1	5	5	4
3	5	1	2	3	3
5	2	3	4	4	4
5	5	1	5	4	5
5	5	5	5	4	5
3	4	3	4	4	3
5	3	1	4	5	5
1	4	1	1	5	3
5	4	5	4	4	5
5	3	4	5	3	4
4	5	4	4	4	3
2	4	2	5	4	4
2	2	2	4	2	4
3	4	2	5	3	4
5	4	2	5	4	3
4	3	4	3	4	4
5	4	2	1	5	4
4	3	5	4	4	4
2	3	2	4	3	2
4	5	4	5	4	4
5	2	2	3	3	4
5	5	4	4	4	3
3	4	2	4	1	3
4	4	3	3	4	4
4	4	2	5	2	4
5	5	5	3	3	2
5	4	4	2	3	3
3	2	2	4	4	4
5	5	1	2	3	2
2	3	4	3	3	2
4	5	5	4	5	5
4	3	2	4	3	4
2	2	1	5	2	1
4	5	5	4	5	5
2	5	5	3	1	2
3	3	2	4	4	4
4	3	4	3	4	5
3	2	3	2	1	4
5	4	1	4	2	1
5	4	5	5	4	4
5	2	5	5	4	4

2	1	1	4	2	3
5	5	3	5	1	2
5	2	5	5	4	5
5	5	3	5	2	5
5	5	5	4	5	5
4	2	3	1	2	2
5	5	3	5	5	5
4	1	1	2	1	1
5	4	2	5	4	5
3	5	2	5	2	3
5	5	1	4	3	3
4	5	2	5	5	4
4	1	1	5	4	3
4	3	5	4	3	2
4	3	1	5	3	2
4	5	4	4	5	5
5	4	3	4	4	4
3	1	4	5	5	4
5	4	2	2	2	4
4	4	5	4	1	5
3	1	1	5	5	3
5	4	3	5	5	5
3	3	1	4	3	3
2	1	3	1	2	1
5	3	2	3	4	3
5	3	4	4	3	5
4	4	2	5	1	5
5	2	3	4	1	3
4	2	2	5	3	3
5	2	3	4	3	4
5	4	5	5	5	5
1	5	2	4	4	3
3	4	1	4	4	5
5	3	2	4	5	4
1	3	1	5	2	2
5	3	5	2	4	5
5	4	4	5	5	5
5	4	5	5	5	4
4	3	4	4	5	5
5	4	4	5	5	5
3	5	1	5	3	4
5	4	5	5	4	5
4	4	2	5	4	4
5	2	3	3	4	5
4	3	5	2	3	4
5	3	3	4	4	5
5	4	5	1	2	4
3	5	2	4	4	4
5	2	5	5	3	5
4	2	1	5	4	5

5	4	5	5	4	3
4	4	4	3	3	4
3	5	4	5	3	4
4	5	4	5	3	4
3	4	2	4	3	5
5	5	2	5	5	4
5	2	5	5	5	5
5	2	1	5	3	4
5	2	5	4	3	3
5	4	5	4	3	3
5	2	2	2	3	5
4	3	2	5	5	5
5	3	4	2	4	5
5	2	5	3	3	3
5	5	3	4	5	3
5	2	3	4	5	4
5	5	3	2	3	5
4	4	5	3	3	1
5	4	5	4	3	4
4	2	4	3	5	3
4	3	5	3	3	4
5	2	5	5	5	2
5	5	4	3	4	5
4	3	2	4	4	2
5	3	5	3	1	3
5	5	5	5	5	5
5	4	3	3	2	3
3	1	2	5	4	5
3	1	2	4	3	2
5	4	4	3	4	5
4	2	3	2	2	4
4	4	4	5	4	3
5	3	4	3	2	4
5	5	5	5	5	5
4	4	3	5	3	3
3	5	5	5	2	5
2	5	2	4	2	3
3	4	2	3	2	2
3	3	2	3	2	4
4	5	2	5	3	3
5	5	5	5	4	5
2	4	5	3	2	3
3	4	2	4	4	4
5	2	1	5	4	4
4	5	5	1	2	1
5	5	1	3	4	5
4	5	1	5	5	5
5	3	5	2	3	3
4	2	1	5	5	3
2	5	1	4	1	4

5	5	2	3	4	5
4	4	4	4	3	3
4	5	1	5	3	2
5	4	3	4	5	5
5	3	2	4	4	3
3	1	1	4	1	2
1	5	1	5	5	5
5	5	2	5	4	5
4	3	3	4	3	5
4	5	4	4	3	4
4	4	3	4	5	4
3	4	4	3	3	4
5	4	5	2	3	3
5	4	5	4	3	5
5	4	3	4	2	3
5	2	1	5	1	5
5	3	5	5	5	5
4	4	5	5	3	4
5	4	3	5	4	5
5	5	4	5	5	5
3	4	2	4	1	5
5	1	5	1	2	3
4	2	1	2	4	4
4	3	1	2	1	3
2	5	1	5	5	5
4	5	3	5	2	1
2	5	1	3	2	5

T0_MOT_18_1	T0_MOT_19_1	T0_MOT_20_1	T0_MOT_21_1	T0_MOT_22_1	T0_MOT_23_1
4	5	3	4	4	5
5	5	4	3	5	5
5	2	2	3	5	5
2	3	5	5	3	3
5	5	5	4	5	5
5	4	2	1	5	5
5	4	1	1	5	4
5	2	2	2	4	4
5	4	3	2	5	5
5	4	4	3	3	5
3	4	5	5	4	5
3	5	5	1	3	4
5	5	5	2	5	4
5	5	4	4	5	5
4	5	4	4	4	5
5	5	5	3	4	3
4	5	3	2	4	3
4	2	3	2	4	2
5	4	4	2	5	5
5	2	3	2	4	5
5	5	3	3	4	4
5	5	3	1	5	1
3	4	4	4	3	5
5	3	4	3	4	5
5	5	4	3	5	5
4	3	3	3	4	5
2	3	4	3	2	4
5	3	2	2	5	5
5	1	1	1	5	4
5	4	1	1	5	3
5	5	1	1	5	2
5	3	4	1	5	3
5	4	5	2	5	4
5	3	5	2	5	5
4	1	1	1	4	4
4	2	1	1	2	4
5	4	5	3	5	5
5	5	2	1	4	3
4	2	2	2	5	5
3	2	2	1	2	5
4	3	3	1	5	5
5	3	4	1	5	4
2	1	1	1	2	5
5	4	5	3	5	5
5	5	5	2	5	5
5	2	3	1	5	5
4	5	5	3	5	5
5	5	4	5	5	5
5	4	3	3	5	5

2	2	1	1	2	5
5	4	5	5	5	4
3	4	3	2	3	5
3	4	2	3	2	5
5	4	3	3	5	5
1	3	1	1	1	2
3	3	3	4	4	3
5	5	4	3	4	5
5	5	5	4	5	5
4	3	2	2	5	5
2	5	4	2	2	5
5	3	1	1	5	5
2	2	2	2	1	5
3	5	3	3	3	3
4	3	5	1	3	4
5	3	3	2	5	5
5	2	2	2	4	5
1	3	1	1	2	1
3	2	4	3	3	5
4	5	5	4	5	5
1	3	1	3	1	5
5	5	5	1	5	4
5	5	5	5	5	5
4	4	4	3	5	5
5	4	4	4	5	5
4	5	2	2	4	4
4	4	5	3	4	5
5	4	4	3	4	4
5	4	4	3	5	4
5	2	5	3	5	5
4	2	4	3	5	3
5	2	5	5	5	5
5	2	3	2	5	4
1	5	5	3	1	5
4	3	3	2	5	5
4	5	5	4	5	5
3	3	4	2	5	5
1	4	2	1	1	5
4	1	5	2	5	5
5	3	1	1	5	4
3	4	2	1	3	5
5	5	4	4	5	5
5	5	5	1	5	5
3	5	5	4	5	5
4	2	2	3	2	4
5	4	3	2	5	5
4	3	2	2	4	4
5	4	4	3	5	5
2	2	5	3	2	4
4	1	3	1	5	2

3	1	2	1	3	4
5	3	4	1	1	4
5	5	3	2	5	4
1	4	1	1	1	1
3	4	1	1	3	1
3	5	4	3	2	4
1	3	4	4	1	5
4	3	5	3	4	4
5	4	5	3	5	4
3	2	3	1	5	5
5	3	2	1	3	2
2	4	5	3	3	4
1	5	5	1	1	5
5	4	5	2	5	5
3	4	4	2	3	4
4	5	1	1	5	5
5	1	1	1	4	2
3	2	3	5	4	4
3	3	5	5	5	5
4	5	2	2	4	4
5	4	4	2	5	4
5	1	1	2	5	3
5	3	2	1	5	4
5	4	1	1	5	4
4	4	4	2	3	5
3	3	2	4	3	2
4	5	5	4	4	5
2	4	1	1	2	2
2	2	3	4	3	5
3	2	2	1	2	2
5	3	2	2	5	5
4	1	3	3	5	4
5	4	3	4	5	5
3	4	3	1	3	4
2	3	5	3	1	5
1	4	3	3	4	4
3	4	4	2	5	4
1	4	1	1	1	4
2	3	5	1	2	5
4	4	3	3	5	5
2	2	4	5	4	2
5	3	3	1	4	5
3	5	3	3	5	5
3	4	3	2	1	2
5	4	1	2	4	3
5	2	4	3	5	5
5	2	3	3	4	5
5	1	5	1	5	5
5	5	4	5	3	5
5	5	5	4	5	5

5	4	4	2	5	5
5	3	5	5	5	5
5	1	3	5	5	5
5	5	3	3	5	4
5	5	5	3	5	5
5	5	3	1	4	3
5	4	5	5	5	5
3	5	2	3	2	4
5	3	4	4	5	5
4	2	4	1	4	4
3	4	4	4	4	5
5	5	4	2	5	5
4	2	2	3	4	5
2	5	3	5	4	5
5	1	3	2	5	5
5	3	4	3	5	5
5	5	3	4	5	4
5	1	3	4	5	4
5	4	3	3	5	4
4	4	5	5	5	5
5	1	4	1	5	2
3	5	4	2	5	5
4	2	2	1	5	3
5	2	3	4	5	3
5	3	3	2	5	4
3	4	4	4	3	4
5	1	4	1	5	5
4	5	5	3	4	5
4	2	1	1	4	4
4	4	2	2	3	5
4	5	5	4	3	5
5	1	4	1	5	3
5	3	3	2	5	4
5	4	2	1	5	1
3	2	1	1	4	3
4	5	5	5	5	5
5	5	5	4	5	5
5	4	5	3	5	5
5	5	5	4	5	5
4	3	5	4	5	4
5	5	3	1	4	5
5	5	4	5	5	4
5	2	4	2	4	5
4	5	3	3	4	5
4	3	4	3	4	5
5	5	4	3	5	4
5	4	3	2	5	5
5	5	4	1	3	5
5	5	3	5	5	5
3	4	2	3	3	4

5	5	1	1	5	5
5	3	3	4	4	5
4	2	4	2	4	5
5	5	4	4	4	5
5	5	4	1	5	5
4	4	5	3	5	5
5	5	3	2	5	5
3	2	1	3	4	5
5	5	5	3	5	5
4	5	5	3	5	5
5	3	2	2	5	4
4	4	3	2	4	4
4	2	5	2	3	5
5	5	5	2	5	5
4	4	5	3	5	4
5	4	1	3	5	4
4	2	3	4	4	5
2	3	3	4	5	3
5	3	3	2	5	4
2	4	5	4	4	5
4	2	1	2	5	5
4	5	5	4	4	5
3	5	5	4	4	5
3	4	2	2	3	2
5	5	2	5	5	5
5	1	2	3	5	5
2	5	4	4	5	4
5	4	2	1	5	4
2	1	1	1	2	3
5	4	5	4	5	5
4	2	3	3	4	5
5	3	4	3	5	4
4	5	4	4	5	5
5	5	5	4	4	5
2	4	5	4	3	5
4	3	5	3	5	5
3	5	4	2	3	3
3	2	2	4	3	5
3	5	3	3	4	3
4	5	5	3	4	5
5	4	3	4	4	5
5	4	5	2	4	4
5	3	4	3	4	5
5	4	3	2	5	3
3	4	5	4	2	4
4	5	3	5	5	5
4	5	4	1	5	3
3	5	2	5	3	5
3	4	2	5	3	5
4	3	2	3	4	4

4	5	3	1	3	5
1	4	3	3	2	3
1	4	2	1	1	4
5	5	4	3	5	5
5	5	5	3	5	5
5	5	3	1	5	5
1	1	1	1	1	5
5	1	2	1	5	4
4	3	4	4	4	4
4	4	3	3	4	4
3	4	4	4	5	3
3	5	3	1	5	3
3	4	5	5	2	5
3	5	5	2	4	5
3	3	2	4	4	5
4	2	1	5	4	5
3	4	5	3	5	5
4	3	4	3	5	5
3	4	5	4	5	5
2	5	5	5	5	5
5	1	3	1	5	5
2	3	5	5	3	5
1	5	2	4	1	4
2	5	3	3	4	2
4	2	3	1	1	3
4	3	4	1	3	4
5	2	1	1	5	2

T0_MOT_24_1	T0_MOT_25_1	T0_MOT_26_1	T0_MOT_27_1	T0_MOT_28_1	T0_MOT_29_1
5	3	5	3	5	5
3	2	3	3	1	5
5	3	2	5	2	5
4	5	5	3	5	5
5	3	5	5	4	5
3	1	2	4	1	5
5	1	2	4	1	5
4	2	2	3	2	5
4	2	4	4	2	5
5	1	4	4	1	5
5	3	5	4	4	5
4	2	4	1	1	5
5	2	5	5	2	5
4	3	5	4	4	5
5	3	4	2	1	5
4	5	5	2	3	5
4	2	2	3	3	5
5	2	3	4	1	5
5	1	3	4	1	5
5	2	4	3	1	5
5	1	2	3	1	5
5	1	2	5	1	5
5	5	5	5	4	5
4	3	4	4	2	4
5	2	5	3	1	5
5	4	5	3	3	5
3	2	3	2	2	3
4	4	3	4	3	5
3	1	1	3	1	5
3	1	1	4	1	5
5	1	5	4	1	5
5	1	4	3	1	5
4	1	4	5	2	5
5	2	5	4	2	5
2	1	1	1	1	4
5	1	1	3	1	5
5	2	5	3	3	5
4	1	1	2	1	5
4	1	2	3	2	5
4	1	3	2	1	2
5	1	3	5	1	5
4	1	4	4	1	5
2	1	1	4	1	5
5	5	4	4	5	5
5	2	5	5	1	5
5	1	3	2	2	5
5	3	5	4	3	5
5	2	2	5	2	5
4	2	5	3	4	5

5	2	1	1	2	3
5	4	4	1	4	4
4	1	3	3	2	5
5	4	3	3	2	5
5	5	5	4	5	5
1	1	1	5	1	5
5	3	3	1	3	5
5	2	3	4	1	4
5	4	5	4	4	5
5	2	4	2	2	5
3	1	5	3	2	5
5	1	2	3	1	5
5	1	3	2	1	5
3	2	3	3	2	5
5	1	4	3	1	5
5	2	4	1	1	5
5	1	4	5	1	5
2	1	1	1	1	3
4	1	3	2	1	2
5	3	5	4	1	5
5	4	2	3	2	5
4	3	4	4	3	5
5	5	5	5	5	5
5	1	4	2	1	5
4	4	5	4	3	5
4	2	1	3	2	5
5	4	5	5	4	5
4	3	5	4	3	5
4	1	4	4	1	4
5	3	4	1	1	5
5	2	3	4	3	5
5	1	5	4	1	5
4	2	4	3	1	3
5	4	5	5	3	5
4	5	5	4	1	4
5	3	5	4	3	4
5	2	4	5	1	5
4	1	2	4	1	4
5	1	3	1	1	4
4	2	1	3	1	5
4	1	2	3	1	5
4	4	4	4	3	5
5	4	5	5	1	5
5	3	5	5	3	5
2	3	2	3	2	4
5	2	3	4	2	5
4	1	2	4	2	3
4	1	5	5	2	4
5	1	5	3	1	4
3	1	5	1	1	5

5	1	3	2	1	3
5	1	2	5	1	4
5	2	3	3	2	5
3	1	1	2	1	2
4	1	1	1	1	5
5	3	5	5	2	5
4	3	5	5	2	3
4	2	5	1	1	4
5	2	5	4	2	5
5	1	4	1	1	4
2	1	2	3	1	5
5	2	4	3	2	5
5	4	4	1	1	5
5	2	5	5	2	5
4	2	4	4	1	5
3	1	1	5	1	5
3	1	3	2	1	5
4	5	5	1	5	5
5	3	5	4	2	5
4	2	2	3	1	5
5	2	4	4	2	5
3	1	2	2	1	3
4	1	2	2	1	5
5	1	1	5	1	5
3	1	4	3	2	5
3	4	3	1	4	5
5	2	5	3	3	5
5	1	1	1	1	4
5	3	3	4	2	5
5	2	3	2	1	5
4	1	2	5	1	5
4	1	3	3	1	5
3	2	4	4	2	5
5	1	3	2	1	5
3	3	5	5	4	3
3	4	3	4	3	4
5	2	4	3	2	5
5	2	1	1	3	5
3	2	4	2	1	3
5	2	5	3	2	5
3	2	3	2	3	4
5	1	3	2	2	5
5	2	5	3	2	5
3	2	4	1	1	3
2	1	2	1	1	4
4	3	4	4	2	4
4	3	2	2	3	5
4	1	4	4	1	5
5	1	5	5	4	5
5	3	5	3	4	5

3	2	4	4	2	5
5	4	2	1	5	5
5	5	5	2	3	5
5	1	2	2	2	5
5	5	5	4	5	5
5	1	3	5	1	5
5	5	5	4	5	5
3	5	4	1	2	5
5	1	5	4	1	5
5	1	3	2	1	5
5	1	3	1	1	5
5	1	5	5	1	5
3	1	1	4	1	5
4	2	3	1	2	5
5	1	5	2	1	5
5	2	5	3	2	5
5	4	2	2	2	5
3	4	3	2	2	5
4	3	3	2	3	5
1	1	5	5	1	5
4	1	3	3	1	5
5	2	5	4	2	5
4	1	2	3	1	5
2	1	1	1	1	4
5	3	4	1	3	4
3	3	4	5	3	4
4	1	5	3	1	5
5	4	4	3	2	5
5	1	1	1	1	5
5	2	4	1	3	4
5	4	5	5	4	3
5	1	2	4	1	5
4	1	5	3	1	5
5	1	2	5	1	5
4	1	1	2	1	5
5	5	5	4	5	5
4	2	5	5	4	5
5	5	4	5	1	4
5	5	5	4	3	5
5	3	5	5	4	5
5	1	5	1	1	5
5	4	4	5	4	5
5	3	4	3	2	5
4	3	3	2	2	4
3	2	3	1	2	5
5	3	4	4	3	5
5	1	4	1	2	5
5	1	3	4	1	5
5	5	5	4	5	5
5	1	5	5	2	5

5	1	3	5	2	5
4	3	3	5	3	5
3	2	4	1	1	5
5	4	3	3	3	5
4	1	3	4	1	5
1	3	5	1	3	5
5	2	5	3	2	5
5	1	2	1	1	4
4	2	5	4	1	4
4	1	5	4	1	5
2	1	1	2	1	5
5	2	4	3	2	5
5	1	3	3	2	5
4	1	5	4	1	5
5	3	4	3	2	5
5	4	3	3	3	5
2	3	4	3	2	5
5	5	4	3	2	1
4	4	4	3	2	5
5	3	4	2	3	5
4	1	1	4	2	4
5	1	5	1	2	5
4	4	5	3	4	5
4	2	2	2	2	4
5	2	2	1	1	5
4	2	4	3	1	5
5	2	3	2	1	5
3	1	2	4	3	5
4	1	2	2	1	4
4	3	5	4	3	5
5	3	3	4	2	3
4	2	3	2	1	5
5	4	4	4	2	5
5	5	5	5	5	5
5	3	4	2	3	5
5	3	5	3	2	1
5	1	3	1	1	4
4	2	2	2	2	4
4	4	3	1	1	5
5	2	4	4	5	5
4	3	3	4	3	5
2	3	5	4	1	5
5	3	3	4	1	5
5	2	3	1	1	5
5	1	4	5	2	5
5	3	3	4	1	5
4	1	2	2	1	5
5	1	1	3	1	5
5	3	3	4	2	5
5	2	4	3	2	4

4	1	4	3	1	5
5	2	2	1	3	3
5	1	3	3	1	5
5	4	4	2	2	5
5	1	5	2	1	4
4	2	4	3	2	5
5	1	5	1	1	1
5	1	4	1	1	5
5	2	5	3	3	5
5	2	3	1	1	5
5	1	4	3	2	5
4	1	3	3	1	5
3	1	4	1	4	4
5	4	5	3	4	5
5	5	3	4	4	5
5	5	4	1	4	5
5	1	5	5	1	5
5	2	3	5	3	4
5	3	5	3	4	5
4	1	4	5	2	5
3	1	2	5	1	4
2	5	5	2	5	1
4	5	2	1	4	5
5	2	3	1	1	5
4	1	3	1	1	5
5	1	2	3	1	4
1	1	1	1	1	5

T0_MOT_30_1	T1_MOT_1	T1_MOT_2	T1_MOT_3	T1_MOT_4	T1_MOT_5
5	5	5	4,00	3,00	5,00
3	4	5	2,00	3,00	5,00
3	5	5	3,00	5,00	3,00
3	5	4	5,00	5,00	5,00
5	4	4	3,00	4,00	3,00
3	5	5	5,00	5,00	5,00
5	5	5	4,00	5,00	5,00
3	4	3	2,00	5,00	1,00
5	5	5	3,00	5,00	5,00
3	5	5	3,00	4,00	2,00
2	5	5	3,00	5,00	5,00
2	5	4	2,00	4,00	5,00
5	3	5	3,00	5,00	5,00
4	5	5	3,00	4,00	5,00
4	5	5	1,00	4,00	2,00
3	5	4	3,00	3,00	1,00
4	4	3	1,00	4,00	2,00
2	5	5	2,00	4,00	3,00
4	5	5	2,00	4,00	4,00
3	5	3	2,00	3,00	2,00
2	4	3	1,00	5,00	5,00
4	3	5	3,00	5,00	5,00
5	5	5	3,00	5,00	5,00
4	5	5	4,00	3,00	3,00
4	5	5	3,00	1,00	4,00
4	4	5	4,00	2,00	1,00
2	3	2	2,00	4,00	5,00
2	5	4	2,00	3,00	2,00
2	3	5	2,00	3,00	1,00
2	5	3	1,00	3,00	1,00
3	3	5	2,00	5,00	5,00
2	2	4	1,00	5,00	3,00
5	4	5	3,00	5,00	5,00
5	5	3	2,00	4,00	3,00
1	2	5	1,00	3,00	1,00
2	3	2	1,00	3,00	3,00
5	5	5	4,00	5,00	5,00
4	4	3	1,00	3,00	4,00
1	5	3	4,00	4,00	2,00
1	5	2	1,00	3,00	4,00
5	5	4	5,00	4,00	4,00
1	3	5	1,00	5,00	1,00
4	3	2	2,00	3,00	3,00
5	5	4	1,00	5,00	4,00
5	5	4	4,00	4,00	3,00
3	5	3	2,00	4,00	2,00
4	5	5	5,00	5,00	5,00
5	5	5	2,00	4,00	5,00
5	5	3	5,00	5,00	5,00

2	5	3	2,00	2,00	2,00
5	4	4	3,00	4,00	3,00
3	3	4	2,00	4,00	3,00
3	5	4	3,00	4,00	5,00
5	5	5	3,00	5,00	5,00
1	2	4	1,00	5,00	1,00
2	4	4	3,00	5,00	5,00
1	5	5	3,00	5,00	1,00
3	4	5	3,00	4,00	5,00
2	5	5	3,00	4,00	2,00
3	5	3	2,00	5,00	1,00
2	5	4	1,00	5,00	3,00
5	5	4	2,00	5,00	5,00
2	3	4	1,00	5,00	5,00
4	5	4	5,00	4,00	4,00
5	3	4	3,00	5,00	3,00
3	4	4	2,00	3,00	4,00
1	2	2	1,00	1,00	2,00
1	5	1	3,00	1,00	1,00
5	5	5	3,00	4,00	5,00
5	3	2	3,00	4,00	3,00
4	5	5	4,00	5,00	5,00
5	5	5	5,00	5,00	5,00
5	5	4	2,00	4,00	4,00
3	4	4	3,00	4,00	3,00
3	3	4	3,00	5,00	4,00
4	4	4	4,00	4,00	5,00
4	4	5	3,00	5,00	5,00
1	5	5	3,00	4,00	3,00
3	5	4	2,00	2,00	3,00
5	3	4	2,00	2,00	3,00
2	5	5	4,00	4,00	5,00
2	4	2	2,00	5,00	3,00
4	5	1	3,00	5,00	2,00
1	5	3	5,00	2,00	1,00
4	5	5	4,00	5,00	5,00
3	5	5	3,00	4,00	5,00
2	5	5	2,00	1,00	2,00
1	5	2	2,00	4,00	1,00
2	4	4	2,00	5,00	4,00
5	5	5	2,00	5,00	5,00
4	4	4	4,00	4,00	3,00
1	5	4	5,00	2,00	2,00
3	4	5	4,00	5,00	5,00
5	4	4	2,00	3,00	2,00
4	3	4	2,00	4,00	2,00
3	4	2	1,00	2,00	1,00
2	5	5	4,00	4,00	4,00
5	5	1	3,00	5,00	1,00
2	3	1	4,00	2,00	1,00

2	5	1	2,00	3,00	1,00
2	5	3	1,00	5,00	2,00
4	3	2	2,00	1,00	3,00
1	4	4	1,00	3,00	1,00
3	4	2	1,00	3,00	2,00
5	3	4	5,00	4,00	5,00
5	3	1	5,00	5,00	5,00
4	3	1	5,00	4,00	1,00
5	4	2	5,00	5,00	3,00
2	5	5	3,00	1,00	1,00
1	5	5	2,00	1,00	5,00
5	5	1	3,00	2,00	2,00
5	4	5	4,00	5,00	5,00
5	5	5	2,00	2,00	1,00
5	3	2	3,00	4,00	2,00
5	5	5	1,00	4,00	3,00
1	4	3	2,00	3,00	1,00
2	5	3	5,00	2,00	5,00
5	5	5	5,00	5,00	4,00
2	5	5	2,00	5,00	5,00
5	4	3	2,00	3,00	2,00
2	5	4	3,00	3,00	2,00
2	3	3	3,00	3,00	3,00
5	5	2	1,00	5,00	2,00
5	4	3	2,00	5,00	4,00
1	5	4	4,00	4,00	3,00
5	5	4	5,00	3,00	3,00
2	4	5	2,00	4,00	4,00
5	5	4	4,00	5,00	3,00
3	4	5	3,00	3,00	3,00
5	5	5	3,00	5,00	4,00
3	4	3	3,00	5,00	3,00
3	5	3	3,00	5,00	3,00
3	5	3	2,00	4,00	5,00
1	5	2	5,00	5,00	4,00
4	3	4	2,00	3,00	5,00
5	5	4	3,00	4,00	2,00
2	3	5	1,00	5,00	5,00
2	4	2	3,00	4,00	1,00
5	5	5	3,00	1,00	2,00
5	4	5	2,00	4,00	3,00
1	2	5	3,00	4,00	1,00
5	5	5	3,00	4,00	5,00
4	4	4	5,00	3,00	2,00
4	5	3	2,00	4,00	2,00
3	3	4	5,00	4,00	4,00
4	5	5	4,00	3,00	2,00
4	4	5	1,00	3,00	1,00
5	3	3	4,00	5,00	3,00
5	4	4	4,00	3,00	3,00

4	4	4	3,00	1,00	3,00
3	5	4	3,00	5,00	3,00
5	4	3	5,00	2,00	1,00
3	5	5	3,00	2,00	1,00
5	5	5	5,00	5,00	5,00
5	2	3	3,00	1,00	2,00
3	5	4	3,00	5,00	5,00
3	4	5	4,00	3,00	4,00
5	5	5	5,00	5,00	5,00
4	5	2	3,00	4,00	2,00
1	5	4	2,00	3,00	3,00
5	5	5	4,00	5,00	2,00
5	5	4	3,00	1,00	2,00
2	2	5	5,00	4,00	5,00
1	5	3	3,00	4,00	2,00
4	4	5	3,00	4,00	3,00
4	5	5	4,00	4,00	5,00
3	5	5	5,00	4,00	5,00
4	5	4	3,00	2,00	4,00
4	5	4	3,00	2,00	4,00
3	4	5	1,00	2,00	1,00
5	5	4	2,00	3,00	4,00
2	5	3	1,00	2,00	1,00
2	4	4	2,00	1,00	1,00
3	5	3	2,00	3,00	2,00
5	3	3	2,00	4,00	2,00
3	5	3	4,00	2,00	2,00
5	4	5	4,00	5,00	5,00
4	4	4	2,00	1,00	2,00
1	3	2	4,00	2,00	5,00
5	3	3	4,00	2,00	5,00
5	4	3	2,00	5,00	3,00
5	5	3	3,00	3,00	4,00
5	5	5	3,00	5,00	3,00
2	4	2	1,00	2,00	3,00
2	5	5	5,00	2,00	4,00
2	5	4	3,00	2,00	3,00
4	5	5	5,00	2,00	5,00
5	5	5	5,00	3,00	5,00
5	5	4	5,00	4,00	4,00
5	5	4	2,00	5,00	1,00
4	5	5	5,00	5,00	5,00
2	5	5	2,00	4,00	1,00
3	5	5	4,00	3,00	1,00
4	4	4	5,00	4,00	3,00
4	5	5	4,00	3,00	1,00
5	4	3	3,00	2,00	1,00
4	5	5	3,00	4,00	4,00
2	5	4	4,00	4,00	4,00
4	5	5	2,00	3,00	4,00

3	5	4	4,00	5,00	2,00
3	4	5	3,00	4,00	3,00
5	4	2	4,00	4,00	4,00
5	5	3	4,00	3,00	2,00
4	5	5	2,00	3,00	5,00
2	5	4	1,00	5,00	1,00
5	4	5	5,00	2,00	5,00
2	5	3	2,00	3,00	3,00
2	5	4	5,00	3,00	4,00
2	4	3	5,00	5,00	4,00
2	4	5	3,00	4,00	3,00
4	4	3	2,00	3,00	4,00
5	5	4	4,00	3,00	4,00
2	5	5	5,00	5,00	5,00
2	5	5	5,00	2,00	5,00
4	4	5	3,00	4,00	5,00
4	2	3	4,00	5,00	3,00
5	5	3	5,00	5,00	5,00
5	5	5	5,00	2,00	3,00
3	5	4	5,00	5,00	3,00
2	4	4	5,00	3,00	3,00
5	5	5	5,00	4,00	4,00
3	5	5	4,00	5,00	5,00
3	3	3	1,00	3,00	5,00
2	5	5	5,00	5,00	4,00
5	5	5	4,00	5,00	1,00
3	5	4	3,00	5,00	2,00
5	5	5	4,00	4,00	5,00
1	5	4	3,00	2,00	2,00
4	4	5	4,00	2,00	3,00
4	4	3	2,00	2,00	3,00
4	5	5	5,00	4,00	5,00
4	3	5	5,00	5,00	4,00
5	5	5	3,00	4,00	5,00
4	5	5	4,00	4,00	4,00
1	5	2	5,00	5,00	5,00
2	5	3	2,00	4,00	3,00
3	4	3	2,00	5,00	3,00
3	4	3	3,00	3,00	2,00
2	5	3	2,00	4,00	4,00
5	5	5	3,00	4,00	2,00
5	3	4	4,00	5,00	4,00
4	4	5	3,00	4,00	5,00
3	4	5	5,00	2,00	5,00
1	5	5	5,00	4,00	3,00
5	5	3	2,00	4,00	2,00
2	5	4	2,00	4,00	4,00
1	3	1	1,00	3,00	5,00
4	5	3	1,00	3,00	2,00
3	5	5	3,00	4,00	4,00

5	5	3	2,00	5,00	5,00
3	3	3	2,00	1,00	2,00
3	5	2	2,00	5,00	5,00
3	5	4	3,00	4,00	4,00
3	5	3	4,00	4,00	5,00
5	4	4	3,00	3,00	3,00
5	5	1	1,00	5,00	3,00
4	4	4	2,00	3,00	4,00
1	5	4	4,00	3,00	5,00
4	4	4	3,00	2,00	3,00
5	5	4	5,00	5,00	4,00
3	5	2	3,00	2,00	4,00
2	2	1	5,00	5,00	1,00
4	5	2	5,00	5,00	4,00
2	4	4	5,00	5,00	5,00
4	2	5	5,00	5,00	2,00
5	5	5	5,00	5,00	2,00
4	5	5	4,00	4,00	4,00
5	5	5	3,00	4,00	3,00
5	5	4	5,00	4,00	5,00
1	5	4	4,00	4,00	2,00
1	5	3	5,00	4,00	4,00
5	4	5	4,00	5,00	2,00
2	2	5	2,00	3,00	2,00
4	4	3	1,00	5,00	5,00
3	3	2	2,00	4,00	1,00
5	5	2	2,00	5,00	3,00

T1_MOT_6	T1_MOT_7	T1_MOT_8	T1_MOT_9	T1_MOT_1	T1_MOT_1	T1_MOT_1	T1_MOT_1	T1_MOT_1
3,00	5	4,00	5,00	5,00	3,00	5,00	3,00	4,00
2,00	4	5,00	4,00	3,00	3,00	4,00	2,00	4,00
5,00	5	3,00	1,00	5,00	3,00	5,00	5,00	3,00
5,00	3	1,00	2,00	2,00	3,00	5,00	5,00	5,00
4,00	4	4,00	2,00	4,00	4,00	4,00	4,00	3,00
5,00	5	5,00	5,00	5,00	5,00	3,00	2,00	5,00
3,00	5	5,00	5,00	5,00	5,00	4,00	5,00	4,00
4,00	4	3,00	1,00	4,00	3,00	3,00	4,00	2,00
5,00	5	5,00	4,00	5,00	5,00	5,00	5,00	4,00
4,00	3	5,00	1,00	5,00	5,00	5,00	3,00	1,00
5,00	5	4,00	4,00	5,00	5,00	4,00	5,00	3,00
4,00	5	3,00	1,00	4,00	4,00	3,00	5,00	3,00
5,00	5	5,00	5,00	5,00	5,00	5,00	5,00	4,00
5,00	5	5,00	4,00	5,00	5,00	4,00	4,00	4,00
5,00	5	4,00	5,00	5,00	5,00	5,00	5,00	2,00
1,00	5	4,00	1,00	3,00	4,00	3,00	2,00	3,00
4,00	4	5,00	1,00	4,00	3,00	3,00	3,00	2,00
4,00	3	5,00	3,00	4,00	5,00	4,00	4,00	2,00
5,00	5	5,00	2,00	5,00	5,00	5,00	3,00	2,00
4,00	3	4,00	1,00	4,00	3,00	4,00	3,00	1,00
5,00	5	5,00	3,00	4,00	5,00	5,00	5,00	2,00
4,00	5	5,00	5,00	5,00	5,00	2,00	2,00	2,00
5,00	5	5,00	5,00	5,00	5,00	5,00	5,00	5,00
4,00	5	4,00	1,00	5,00	4,00	3,00	2,00	4,00
5,00	4	5,00	5,00	5,00	4,00	5,00	3,00	5,00
4,00	2	4,00	1,00	4,00	2,00	3,00	1,00	3,00
1,00	1	1,00	4,00	3,00	2,00	3,00	5,00	2,00
4,00	5	5,00	1,00	4,00	4,00	5,00	2,00	2,00
5,00	5	5,00	2,00	4,00	4,00	2,00	2,00	1,00
4,00	5	5,00	1,00	5,00	5,00	5,00	3,00	2,00
3,00	5	5,00	2,00	5,00	5,00	5,00	5,00	3,00
4,00	4	3,00	1,00	2,00	1,00	4,00	5,00	1,00
4,00	4	5,00	3,00	5,00	5,00	5,00	5,00	2,00
4,00	5	5,00	1,00	5,00	4,00	5,00	4,00	4,00
1,00	4	4,00	1,00	3,00	5,00	3,00	1,00	1,00
5,00	5	5,00	2,00	5,00	4,00	3,00	4,00	2,00
5,00	4	5,00	2,00	5,00	4,00	4,00	5,00	4,00
5,00	5	5,00	4,00	4,00	4,00	2,00	3,00	2,00
5,00	5	5,00	1,00	5,00	3,00	5,00	5,00	4,00
5,00	5	3,00	1,00	5,00	2,00	3,00	2,00	2,00
5,00	4	4,00	1,00	5,00	5,00	5,00	3,00	2,00
1,00	4	5,00	5,00	2,00	3,00	5,00	5,00	1,00
2,00	3	2,00	1,00	4,00	2,00	3,00	3,00	1,00
4,00	5	5,00	2,00	5,00	5,00	5,00	5,00	1,00
5,00	4	5,00	1,00	4,00	4,00	4,00	4,00	3,00
5,00	4	4,00	1,00	5,00	4,00	2,00	3,00	1,00
5,00	5	4,00	3,00	5,00	5,00	5,00	4,00	3,00
5,00	5	5,00	3,00	5,00	5,00	5,00	5,00	2,00
5,00	4	5,00	4,00	5,00	4,00	5,00	5,00	5,00

5,00	3	4,00	2,00	4,00	2,00	5,00	2,00	3,00
4,00	4	4,00	2,00	4,00	4,00	4,00	4,00	3,00
4,00	4	4,00	2,00	4,00	4,00	3,00	3,00	1,00
5,00	4	3,00	2,00	5,00	2,00	5,00	3,00	2,00
4,00	3	5,00	4,00	5,00	4,00	5,00	5,00	3,00
2,00	5	1,00	1,00	3,00	2,00	2,00	5,00	1,00
4,00	4	3,00	1,00	4,00	2,00	3,00	5,00	3,00
5,00	5	5,00	1,00	4,00	5,00	5,00	5,00	4,00
3,00	5	5,00	1,00	4,00	5,00	4,00	4,00	3,00
4,00	4	4,00	1,00	4,00	3,00	5,00	4,00	2,00
2,00	5	3,00	1,00	5,00	3,00	4,00	5,00	1,00
5,00	5	5,00	1,00	5,00	4,00	3,00	4,00	1,00
5,00	3	2,00	1,00	5,00	4,00	4,00	5,00	3,00
1,00	1	1,00	3,00	1,00	1,00	5,00	5,00	5,00
5,00	3	4,00	1,00	5,00	5,00	4,00	3,00	3,00
3,00	5	5,00	1,00	4,00	5,00	4,00	5,00	3,00
5,00	5	5,00	4,00	3,00	4,00	4,00	4,00	2,00
3,00	1	1,00	1,00	1,00	1,00	3,00	1,00	1,00
3,00	5	2,00	1,00	2,00	2,00	3,00	5,00	2,00
4,00	4	5,00	1,00	4,00	4,00	5,00	3,00	2,00
5,00	3	1,00	3,00	3,00	2,00	4,00	5,00	3,00
5,00	4	5,00	1,00	5,00	3,00	4,00	5,00	3,00
5,00	5	4,00	5,00	5,00	5,00	5,00	5,00	5,00
5,00	5	4,00	2,00	5,00	4,00	5,00	4,00	2,00
4,00	4	4,00	2,00	4,00	4,00	4,00	4,00	3,00
3,00	4	3,00	1,00	3,00	4,00	4,00	4,00	3,00
5,00	3	4,00	4,00	4,00	5,00	5,00	5,00	4,00
4,00	3	4,00	3,00	3,00	4,00	5,00	5,00	2,00
3,00	5	5,00	1,00	5,00	5,00	4,00	4,00	3,00
5,00	5	5,00	1,00	4,00	4,00	5,00	4,00	1,00
5,00	3	4,00	2,00	2,00	3,00	4,00	2,00	1,00
5,00	5	5,00	1,00	5,00	5,00	5,00	5,00	5,00
4,00	5	5,00	1,00	4,00	2,00	4,00	5,00	2,00
4,00	5	1,00	1,00	5,00	3,00	3,00	5,00	4,00
5,00	5	5,00	1,00	5,00	2,00	5,00	3,00	1,00
5,00	4	5,00	1,00	5,00	4,00	5,00	5,00	3,00
4,00	5	3,00	1,00	5,00	5,00	5,00	4,00	3,00
5,00	4	2,00	1,00	5,00	4,00	5,00	2,00	2,00
5,00	5	5,00	1,00	5,00	1,00	4,00	2,00	1,00
5,00	5	3,00	1,00	4,00	4,00	4,00	5,00	4,00
5,00	4	4,00	2,00	5,00	5,00	5,00	4,00	1,00
4,00	4	4,00	1,00	4,00	4,00	4,00	3,00	3,00
5,00	4	5,00	1,00	3,00	2,00	3,00	2,00	5,00
4,00	5	5,00	4,00	5,00	3,00	4,00	5,00	3,00
2,00	4	3,00	2,00	4,00	4,00	4,00	4,00	3,00
3,00	3	4,00	1,00	3,00	3,00	2,00	3,00	1,00
4,00	2	2,00	2,00	3,00	2,00	3,00	2,00	1,00
4,00	5	5,00	2,00	3,00	4,00	3,00	3,00	1,00
5,00	5	1,00	1,00	5,00	1,00	4,00	4,00	2,00
2,00	5	5,00	1,00	3,00	2,00	3,00	1,00	1,00

3,00	3	5,00	1,00	5,00	3,00	4,00	4,00	2,00
5,00	4	3,00	1,00	4,00	3,00	3,00	5,00	2,00
5,00	5	5,00	1,00	4,00	5,00	5,00	3,00	2,00
4,00	5	3,00	1,00	4,00	4,00	1,00	3,00	1,00
5,00	4	2,00	1,00	3,00	3,00	4,00	3,00	1,00
5,00	4	3,00	4,00	4,00	5,00	4,00	5,00	4,00
4,00	5	1,00	1,00	3,00	2,00	5,00	5,00	4,00
2,00	4	4,00	1,00	3,00	1,00	3,00	4,00	2,00
5,00	4	3,00	1,00	4,00	3,00	5,00	5,00	3,00
5,00	3	5,00	1,00	5,00	3,00	4,00	3,00	2,00
3,00	1	4,00	5,00	3,00	5,00	3,00	1,00	1,00
5,00	4	4,00	2,00	5,00	4,00	5,00	3,00	3,00
5,00	3	4,00	4,00	4,00	4,00	3,00	5,00	4,00
5,00	5	5,00	1,00	3,00	3,00	1,00	1,00	1,00
3,00	5	4,00	1,00	3,00	2,00	4,00	5,00	2,00
3,00	4	5,00	2,00	4,00	5,00	4,00	2,00	1,00
4,00	2	3,00	4,00	3,00	2,00	1,00	2,00	1,00
5,00	5	4,00	1,00	5,00	5,00	5,00	3,00	5,00
5,00	5	5,00	2,00	5,00	5,00	5,00	5,00	3,00
5,00	4	5,00	2,00	4,00	4,00	5,00	5,00	3,00
5,00	3	3,00	1,00	4,00	3,00	3,00	3,00	1,00
5,00	5	5,00	1,00	5,00	4,00	3,00	3,00	3,00
4,00	4	5,00	1,00	4,00	4,00	3,00	4,00	2,00
5,00	5	5,00	1,00	5,00	2,00	3,00	5,00	1,00
5,00	5	4,00	2,00	4,00	3,00	5,00	5,00	2,00
3,00	3	4,00	2,00	5,00	4,00	5,00	4,00	3,00
5,00	5	4,00	1,00	3,00	4,00	4,00	3,00	4,00
5,00	5	4,00	4,00	4,00	5,00	5,00	4,00	3,00
5,00	5	2,00	2,00	5,00	4,00	4,00	5,00	3,00
4,00	4	4,00	4,00	5,00	5,00	5,00	3,00	3,00
5,00	5	3,00	4,00	5,00	4,00	5,00	5,00	2,00
4,00	4	5,00	1,00	4,00	4,00	4,00	4,00	2,00
5,00	5	5,00	3,00	5,00	4,00	4,00	4,00	4,00
5,00	4	5,00	3,00	4,00	4,00	3,00	4,00	1,00
3,00	4	2,00	2,00	3,00	4,00	5,00	2,00	5,00
3,00	3	2,00	2,00	3,00	3,00	4,00	3,00	4,00
4,00	5	5,00	3,00	5,00	4,00	4,00	5,00	3,00
5,00	5	3,00	2,00	3,00	5,00	5,00	5,00	1,00
2,00	3	3,00	1,00	4,00	2,00	3,00	5,00	3,00
4,00	3	4,00	4,00	4,00	5,00	5,00	2,00	4,00
2,00	3	5,00	3,00	4,00	4,00	4,00	3,00	2,00
3,00	5	3,00	1,00	4,00	5,00	5,00	3,00	1,00
5,00	5	5,00	5,00	5,00	4,00	5,00	3,00	2,00
4,00	2	4,00	1,00	5,00	4,00	3,00	2,00	3,00
4,00	3	5,00	2,00	4,00	3,00	3,00	3,00	2,00
3,00	4	4,00	3,00	3,00	3,00	4,00	4,00	3,00
4,00	3	5,00	2,00	5,00	4,00	3,00	2,00	3,00
2,00	4	5,00	1,00	5,00	3,00	4,00	3,00	3,00
4,00	2	2,00	3,00	5,00	3,00	5,00	5,00	5,00
5,00	5	2,00	4,00	5,00	5,00	5,00	3,00	5,00

4,00	4	5,00	1,00	3,00	4,00	3,00	2,00	3,00
5,00	5	5,00	5,00	5,00	3,00	5,00	5,00	5,00
5,00	5	4,00	2,00	5,00	4,00	5,00	1,00	4,00
4,00	5	5,00	1,00	3,00	5,00	5,00	5,00	4,00
5,00	4	5,00	5,00	5,00	5,00	5,00	4,00	5,00
4,00	4	5,00	2,00	4,00	4,00	4,00	3,00	2,00
5,00	5	5,00	5,00	5,00	5,00	5,00	5,00	5,00
3,00	2	5,00	4,00	1,00	5,00	5,00	1,00	3,00
5,00	5	5,00	5,00	5,00	5,00	5,00	5,00	5,00
4,00	3	4,00	2,00	5,00	2,00	3,00	5,00	1,00
5,00	5	5,00	4,00	4,00	5,00	5,00	5,00	1,00
4,00	4	5,00	5,00	5,00	4,00	5,00	5,00	4,00
3,00	3	4,00	1,00	2,00	2,00	5,00	2,00	1,00
5,00	3	2,00	3,00	4,00	3,00	5,00	2,00	5,00
5,00	5	5,00	3,00	5,00	4,00	3,00	4,00	1,00
5,00	4	5,00	3,00	4,00	3,00	3,00	3,00	3,00
5,00	3	4,00	4,00	5,00	5,00	5,00	4,00	3,00
3,00	4	3,00	1,00	5,00	5,00	4,00	1,00	4,00
5,00	5	4,00	2,00	5,00	4,00	5,00	4,00	3,00
1,00	5	4,00	2,00	5,00	5,00	2,00	1,00	4,00
4,00	5	5,00	1,00	3,00	5,00	1,00	1,00	1,00
5,00	5	3,00	3,00	5,00	5,00	5,00	4,00	2,00
5,00	4	3,00	2,00	4,00	3,00	4,00	3,00	1,00
4,00	2	3,00	1,00	2,00	3,00	3,00	1,00	4,00
5,00	4	5,00	2,00	4,00	4,00	5,00	4,00	1,00
4,00	3	3,00	4,00	4,00	3,00	4,00	5,00	4,00
5,00	4	3,00	3,00	5,00	3,00	4,00	4,00	4,00
2,00	4	4,00	1,00	3,00	3,00	5,00	4,00	4,00
5,00	4	5,00	2,00	4,00	3,00	3,00	1,00	1,00
3,00	3	2,00	1,00	4,00	2,00	3,00	2,00	4,00
5,00	4	5,00	2,00	5,00	4,00	4,00	5,00	5,00
5,00	5	4,00	1,00	4,00	4,00	2,00	4,00	1,00
5,00	5	4,00	1,00	5,00	4,00	5,00	4,00	4,00
4,00	3	5,00	1,00	2,00	5,00	5,00	3,00	4,00
4,00	4	3,00	1,00	5,00	4,00	3,00	5,00	2,00
5,00	4	4,00	4,00	3,00	5,00	5,00	3,00	5,00
5,00	5	5,00	4,00	5,00	5,00	4,00	4,00	4,00
5,00	4	4,00	4,00	5,00	2,00	4,00	1,00	5,00
5,00	4	4,00	5,00	5,00	5,00	5,00	4,00	5,00
5,00	5	5,00	4,00	5,00	4,00	5,00	5,00	5,00
5,00	5	5,00	1,00	4,00	4,00	5,00	5,00	1,00
5,00	5	5,00	5,00	5,00	5,00	4,00	4,00	5,00
5,00	5	4,00	1,00	5,00	4,00	4,00	2,00	2,00
4,00	5	4,00	2,00	5,00	4,00	5,00	4,00	5,00
4,00	4	4,00	2,00	4,00	4,00	3,00	4,00	3,00
4,00	5	4,00	2,00	4,00	5,00	5,00	4,00	3,00
4,00	5	3,00	1,00	3,00	2,00	5,00	3,00	2,00
5,00	4	5,00	3,00	5,00	4,00	5,00	4,00	4,00
4,00	4	4,00	4,00	4,00	5,00	5,00	4,00	5,00
5,00	5	3,00	3,00	4,00	4,00	4,00	3,00	2,00

5,00	4	5,00	3,00	5,00	5,00	4,00	5,00	3,00
4,00	5	5,00	4,00	4,00	3,00	4,00	4,00	5,00
4,00	5	4,00	1,00	4,00	2,00	5,00	4,00	4,00
5,00	5	5,00	3,00	4,00	3,00	5,00	4,00	4,00
5,00	4	5,00	3,00	4,00	4,00	5,00	3,00	1,00
1,00	5	3,00	1,00	2,00	4,00	5,00	5,00	1,00
5,00	5	4,00	2,00	4,00	5,00	4,00	2,00	2,00
5,00	4	4,00	2,00	4,00	3,00	5,00	4,00	3,00
4,00	4	5,00	2,00	4,00	3,00	4,00	3,00	4,00
2,00	5	4,00	1,00	3,00	4,00	3,00	4,00	2,00
3,00	3	5,00	4,00	4,00	3,00	4,00	3,00	3,00
5,00	4	4,00	2,00	4,00	3,00	4,00	2,00	1,00
5,00	4	5,00	3,00	4,00	5,00	5,00	4,00	5,00
5,00	5	4,00	1,00	5,00	5,00	4,00	5,00	4,00
4,00	4	4,00	5,00	5,00	4,00	5,00	3,00	2,00
4,00	5	5,00	4,00	4,00	4,00	4,00	3,00	4,00
2,00	3	2,00	4,00	2,00	5,00	3,00	1,00	2,00
1,00	5	4,00	3,00	2,00	1,00	1,00	3,00	2,00
4,00	3	5,00	5,00	5,00	5,00	5,00	2,00	3,00
4,00	4	4,00	2,00	5,00	4,00	5,00	4,00	5,00
4,00	2	3,00	1,00	3,00	2,00	2,00	3,00	4,00
5,00	5	3,00	5,00	5,00	5,00	5,00	4,00	5,00
4,00	4	3,00	2,00	5,00	1,00	5,00	5,00	4,00
3,00	3	4,00	2,00	4,00	3,00	5,00	4,00	1,00
5,00	4	1,00	3,00	4,00	5,00	5,00	5,00	5,00
4,00	5	5,00	3,00	4,00	4,00	5,00	3,00	4,00
5,00	3	4,00	2,00	3,00	4,00	5,00	4,00	1,00
3,00	4	5,00	3,00	5,00	3,00	5,00	1,00	4,00
4,00	3	4,00	2,00	3,00	2,00	3,00	2,00	1,00
4,00	4	5,00	3,00	4,00	4,00	4,00	3,00	4,00
4,00	3	5,00	1,00	3,00	3,00	4,00	3,00	2,00
4,00	4	4,00	4,00	4,00	4,00	5,00	4,00	5,00
4,00	3	4,00	1,00	4,00	4,00	5,00	3,00	5,00
5,00	5	3,00	2,00	5,00	5,00	5,00	5,00	5,00
5,00	4	5,00	3,00	5,00	5,00	4,00	4,00	4,00
5,00	5	5,00	1,00	3,00	3,00	3,00	5,00	5,00
5,00	3	4,00	1,00	4,00	2,00	2,00	5,00	2,00
4,00	4	3,00	2,00	3,00	2,00	4,00	4,00	3,00
5,00	3	3,00	1,00	2,00	3,00	3,00	4,00	2,00
5,00	5	3,00	1,00	5,00	2,00	3,00	4,00	2,00
5,00	4	5,00	3,00	5,00	4,00	4,00	5,00	3,00
3,00	5	4,00	2,00	4,00	2,00	4,00	3,00	4,00
5,00	4	5,00	4,00	5,00	5,00	4,00	5,00	2,00
4,00	4	5,00	3,00	5,00	4,00	5,00	1,00	3,00
4,00	4	1,00	1,00	4,00	4,00	4,00	4,00	5,00
4,00	5	3,00	2,00	4,00	3,00	5,00	5,00	2,00
3,00	5	5,00	1,00	5,00	3,00	4,00	5,00	2,00
4,00	3	2,00	3,00	4,00	5,00	5,00	3,00	1,00
5,00	4	4,00	3,00	5,00	5,00	4,00	1,00	1,00
5,00	5	4,00	2,00	4,00	4,00	3,00	4,00	4,00

5,00	4	5,00	3,00	5,00	4,00	4,00	3,00	3,00
3,00	3	2,00	2,00	3,00	3,00	4,00	2,00	3,00
5,00	4	1,00	2,00	5,00	3,00	4,00	5,00	2,00
5,00	5	5,00	4,00	4,00	5,00	5,00	5,00	4,00
5,00	5	4,00	1,00	4,00	4,00	5,00	4,00	5,00
3,00	4	3,00	1,00	3,00	3,00	3,00	2,00	2,00
4,00	1	1,00	1,00	5,00	1,00	1,00	5,00	1,00
5,00	5	5,00	3,00	5,00	5,00	5,00	4,00	2,00
5,00	5	4,00	2,00	4,00	4,00	5,00	4,00	4,00
5,00	3	3,00	2,00	2,00	3,00	3,00	2,00	3,00
4,00	4	4,00	2,00	5,00	4,00	4,00	5,00	2,00
4,00	3	4,00	2,00	4,00	2,00	4,00	4,00	3,00
1,00	3	1,00	1,00	2,00	1,00	4,00	3,00	5,00
4,00	5	1,00	1,00	5,00	1,00	5,00	5,00	2,00
4,00	4	3,00	4,00	5,00	4,00	5,00	3,00	5,00
5,00	4	4,00	5,00	1,00	1,00	4,00	2,00	5,00
5,00	5	4,00	1,00	5,00	3,00	5,00	5,00	3,00
5,00	5	4,00	3,00	5,00	5,00	5,00	4,00	5,00
5,00	5	4,00	4,00	5,00	5,00	4,00	3,00	1,00
4,00	3	3,00	1,00	4,00	4,00	5,00	5,00	5,00
3,00	5	4,00	1,00	5,00	2,00	3,00	4,00	1,00
2,00	2	1,00	2,00	1,00	3,00	5,00	1,00	5,00
3,00	4	2,00	1,00	2,00	1,00	5,00	3,00	4,00
4,00	1	3,00	1,00	2,00	5,00	4,00	2,00	1,00
4,00	4	2,00	2,00	4,00	4,00	5,00	5,00	3,00
3,00	3	3,00	1,00	2,00	3,00	3,00	3,00	1,00
2,00	4	5,00	1,00	2,00	2,00	4,00	5,00	2,00

T1_MOT_1	T1_MOT_1	T1_MOT_1	T1_MOT_1	T1_MOT_1	T1_MOT_2	T1_MOT_2	T1_MOT_2	T1_MOT_2
5,00	5,00	5	4	5	3	3	5	3
3,00	5,00	4	5	4	4	3	5	3
5,00	5,00	2	4	2	2	3	3	5
5,00	3,00	4	1	5	5	5	1	5
4,00	3,00	4	4	4	3	2	4	4
5,00	5,00	3	5	4	1	1	5	5
5,00	5,00	5	5	3	5	1	5	3
5,00	2,00	3	3	2	2	2	3	3
5,00	4,00	5	5	3	4	1	5	5
5,00	3,00	3	5	5	2	2	5	5
5,00	4,00	5	4	5	5	3	4	5
5,00	3,00	5	2	4	5	1	4	5
5,00	5,00	5	5	3	5	2	5	3
5,00	5,00	5	5	4	3	3	5	5
5,00	5,00	5	5	5	1	1	5	5
3,00	4,00	5	3	5	5	3	3	4
4,00	5,00	5	4	3	2	2	4	4
3,00	4,00	5	4	3	4	2	3	4
3,00	3,00	4	5	4	4	3	5	5
5,00	4,00	3	4	3	3	1	5	2
5,00	5,00	5	5	5	3	1	5	5
5,00	5,00	5	5	5	3	1	5	5
5,00	5,00	5	5	5	5	5	5	5
4,00	3,00	1	4	4	4	2	4	5
5,00	4,00	2	5	5	5	2	5	5
5,00	5,00	2	2	3	4	2	2	5
1,00	2,00	2	2	5	1	2	2	4
3,00	2,00	4	5	4	4	4	4	4
4,00	2,00	5	5	2	2	1	3	4
4,00	4,00	5	5	4	1	1	5	4
3,00	1,00	5	5	5	1	1	5	3
4,00	5,00	5	5	3	4	1	5	4
5,00	5,00	5	5	5	2	2	5	5
3,00	5,00	5	5	1	5	2	5	5
3,00	2,00	3	5	1	1	1	5	1
5,00	3,00	5	5	3	1	1	5	3
5,00	4,00	5	5	4	5	3	5	5
4,00	4,00	3	4	5	1	2	5	3
5,00	2,00	2	5	1	4	2	5	5
5,00	1,00	2	3	2	2	1	2	4
5,00	2,00	3	4	3	5	1	3	5
4,00	3,00	4	5	2	4	4	5	5
2,00	2,00	3	2	1	2	1	2	4
5,00	5,00	5	5	4	5	1	5	5
5,00	4,00	5	5	4	4	3	5	4
5,00	3,00	4	4	2	2	1	4	4
5,00	5,00	4	5	3	4	2	4	5
5,00	5,00	5	5	5	4	1	5	5
5,00	4,00	5	5	5	5	4	5	5

3,00	3,00	2	2	1	1	1	2	5
4,00	3,00	4	4	4	3	2	4	4
3,00	3,00	4	4	4	3	4	4	5
5,00	4,00	4	2	5	2	4	2	3
2,00	5,00	4	3	5	3	5	4	5
1,00	4,00	3	1	5	1	1	1	2
4,00	5,00	4	2	4	3	2	3	2
3,00	4,00	4	5	4	4	1	5	4
3,00	4,00	4	5	5	5	2	5	4
4,00	4,00	4	4	3	2	2	5	4
3,00	3,00	5	3	5	4	2	2	5
5,00	2,00	3	5	2	1	1	5	5
4,00	3,00	5	3	5	5	2	4	5
5,00	5,00	5	1	5	1	3	2	4
4,00	4,00	3	3	4	4	2	4	2
5,00	1,00	5	5	2	2	1	5	4
5,00	5,00	4	5	4	4	2	5	5
1,00	1,00	2	1	1	1	1	2	1
1,00	1,00	1	3	1	5	2	1	4
4,00	3,00	4	4	5	4	5	4	5
4,00	4,00	2	1	3	2	4	1	3
3,00	4,00	5	5	4	5	3	4	5
4,00	5,00	5	5	4	5	5	5	5
5,00	5,00	5	4	5	3	2	4	5
4,00	3,00	4	4	4	3	2	4	4
4,00	3,00	2	2	3	3	1	3	4
4,00	3,00	5	5	4	4	4	5	5
4,00	4,00	4	4	3	3	2	3	4
3,00	3,00	3	5	3	4	2	5	2
5,00	4,00	5	5	5	4	3	5	5
3,00	4,00	2	3	4	2	3	2	3
5,00	5,00	5	5	5	5	5	5	5
4,00	3,00	4	5	3	4	3	5	4
5,00	2,00	5	1	5	5	1	3	5
3,00	3,00	5	5	5	5	3	5	5
5,00	5,00	4	4	4	5	1	4	5
3,00	5,00	5	3	5	4	2	3	5
5,00	5,00	5	2	5	3	2	2	5
5,00	1,00	2	5	1	5	2	5	4
5,00	5,00	4	5	3	1	1	5	5
5,00	4,00	4	4	5	3	2	4	5
3,00	3,00	4	5	3	3	3	5	4
5,00	2,00	5	5	3	4	1	5	5
3,00	2,00	5	5	4	3	2	5	5
2,00	3,00	4	3	2	3	3	3	2
3,00	1,00	4	4	2	3	1	3	4
4,00	2,00	1	2	3	1	1	3	3
4,00	3,00	3	5	4	3	3	5	4
5,00	2,00	5	2	3	4	2	2	5
4,00	2,00	1	5	1	5	1	5	4

5,00	4,00	4	4	2	3	1	5	5
4,00	3,00	2	4	3	3	1	3	4
5,00	5,00	4	5	5	5	2	5	5
3,00	5,00	1	2	5	1	1	2	2
3,00	2,00	4	3	3	1	1	2	1
5,00	3,00	4	3	5	4	4	4	4
5,00	3,00	3	1	5	5	2	1	5
3,00	2,00	3	4	2	5	3	4	3
4,00	4,00	5	3	5	5	4	3	4
4,00	3,00	2	3	4	4	3	4	5
3,00	5,00	3	5	4	1	1	3	5
4,00	4,00	4	3	4	2	2	3	5
5,00	5,00	5	3	5	4	4	4	5
5,00	1,00	5	5	3	5	1	5	5
4,00	3,00	2	3	4	4	2	4	3
3,00	3,00	4	5	2	1	1	4	3
3,00	4,00	3	4	2	2	1	3	3
4,00	3,00	5	4	5	5	5	4	5
5,00	4,00	5	5	3	5	5	5	5
5,00	5,00	4	5	5	1	2	5	5
4,00	2,00	3	4	3	4	1	4	4
5,00	2,00	5	5	2	1	1	5	4
3,00	2,00	3	5	2	2	1	5	3
5,00	4,00	5	4	3	1	1	3	4
5,00	4,00	4	3	5	4	2	3	5
3,00	4,00	3	3	3	3	4	4	5
4,00	4,00	3	4	4	5	5	4	5
4,00	4,00	3	4	5	2	1	3	3
5,00	5,00	4	1	3	4	3	2	5
4,00	3,00	5	4	4	3	3	4	4
5,00	5,00	4	3	4	2	1	5	5
3,00	1,00	3	4	2	3	3	4	4
4,00	3,00	3	5	4	3	2	4	4
4,00	3,00	5	4	1	3	1	3	4
2,00	2,00	1	2	3	5	3	3	5
2,00	3,00	2	3	3	1	3	2	3
4,00	3,00	5	5	3	5	2	5	5
3,00	4,00	5	3	5	1	3	5	5
4,00	1,00	2	2	3	3	1	3	5
5,00	5,00	4	4	4	3	4	5	5
4,00	4,00	3	4	5	2	1	4	4
4,00	2,00	1	4	5	5	5	5	5
4,00	5,00	5	5	5	2	3	5	5
4,00	5,00	5	4	5	5	3	4	3
4,00	3,00	3	5	4	2	1	5	4
3,00	3,00	4	4	3	3	3	4	4
4,00	3,00	4	4	2	5	3	4	5
4,00	3,00	1	5	2	3	1	5	1
5,00	4,00	3	1	2	3	3	2	5
5,00	4,00	4	3	5	2	2	4	5

4,00	3,00	3	5	5	5	3	4	5
5,00	5,00	4	5	4	5	5	5	5
3,00	2,00	3	4	5	5	5	4	5
5,00	2,00	4	5	5	4	4	3	5
5,00	5,00	5	5	5	4	5	5	5
4,00	2,00	3	5	4	1	2	5	4
5,00	5,00	5	5	5	5	5	5	5
2,00	4,00	2	5	5	1	3	3	2
5,00	5,00	4	5	3	5	2	5	5
4,00	2,00	3	4	2	3	1	2	4
5,00	5,00	4	5	1	5	3	4	4
5,00	5,00	4	5	5	4	3	5	4
5,00	4,00	1	2	3	2	4	5	5
3,00	2,00	3	3	5	2	5	4	4
5,00	5,00	2	5	3	4	1	5	4
3,00	5,00	3	4	3	3	2	5	4
4,00	5,00	4	5	4	4	4	5	4
3,00	2,00	3	4	5	5	3	5	5
4,00	4,00	3	4	4	4	3	5	5
3,00	1,00	4	4	5	5	5	5	5
4,00	5,00	4	5	2	1	4	5	4
5,00	5,00	5	4	2	3	2	5	5
4,00	3,00	4	3	2	1	1	2	4
1,00	2,00	1	4	5	1	3	3	4
4,00	3,00	5	4	5	1	1	4	3
4,00	3,00	4	2	4	5	3	4	4
5,00	5,00	5	3	3	3	4	3	5
3,00	4,00	2	5	5	5	3	4	5
5,00	2,00	3	4	1	1	1	3	5
3,00	4,00	3	3	4	5	1	3	4
5,00	4,00	4	4	5	5	4	5	5
4,00	2,00	3	4	1	3	1	4	5
5,00	1,00	3	4	1	3	1	3	5
4,00	1,00	3	5	5	5	1	5	3
5,00	4,00	3	4	3	2	2	4	4
4,00	4,00	5	5	4	5	5	5	5
4,00	5,00	3	5	4	5	3	5	5
5,00	4,00	4	5	5	4	5	5	5
5,00	5,00	4	5	5	5	4	5	5
4,00	4,00	5	5	4	5	4	5	5
5,00	4,00	5	5	5	4	3	5	4
5,00	4,00	5	5	4	5	5	5	4
5,00	4,00	4	4	1	3	2	3	5
5,00	3,00	4	5	4	4	3	5	5
3,00	4,00	4	5	3	3	4	4	5
5,00	5,00	4	5	3	4	4	5	5
3,00	2,00	2	4	3	3	3	5	4
5,00	5,00	5	5	5	4	3	4	4
4,00	4,00	4	4	4	4	5	4	4
4,00	3,00	5	3	5	4	2	3	4

5,00	5,00	5	5	5	4	1	2	5
4,00	4,00	5	4	4	4	4	4	5
5,00	3,00	4	4	1	4	2	4	5
4,00	5,00	4	5	5	4	3	5	5
4,00	3,00	5	4	5	4	2	4	4
5,00	5,00	5	4	4	5	5	4	5
2,00	4,00	4	5	5	5	5	5	5
5,00	5,00	4	4	4	3	2	2	5
3,00	3,00	4	5	4	5	2	4	5
5,00	4,00	5	4	3	5	1	3	5
4,00	4,00	4	5	4	3	3	5	4
5,00	3,00	3	3	4	3	3	4	4
5,00	4,00	5	5	5	4	5	5	5
5,00	5,00	3	4	5	5	3	3	5
4,00	5,00	4	4	5	5	3	5	5
5,00	5,00	4	4	4	3	3	4	4
4,00	3,00	4	2	3	1	3	4	2
4,00	5,00	4	4	4	4	5	5	5
4,00	4,00	4	5	5	5	2	5	4
5,00	5,00	4	4	5	4	4	4	5
4,00	4,00	4	3	4	2	1	2	4
5,00	4,00	4	3	5	5	5	4	5
4,00	5,00	5	3	5	3	2	2	5
3,00	4,00	3	3	3	2	1	2	2
4,00	2,00	4	5	5	5	5	5	5
5,00	5,00	4	5	3	4	4	4	5
3,00	3,00	4	4	5	2	3	1	2
5,00	4,00	5	5	3	3	3	4	4
4,00	3,00	3	3	2	2	2	4	4
2,00	4,00	4	5	3	4	4	4	4
2,00	2,00	5	5	2	1	1	4	4
4,00	4,00	4	4	4	4	4	4	5
4,00	4,00	3	4	5	3	2	5	5
5,00	5,00	5	4	5	5	5	4	5
4,00	5,00	5	5	4	5	4	4	5
5,00	3,00	5	5	5	5	2	2	5
5,00	3,00	1	2	2	2	2	3	4
3,00	2,00	2	2	3	2	2	2	3
3,00	2,00	2	2	5	3	3	4	4
5,00	2,00	4	4	3	2	5	5	5
3,00	5,00	4	5	5	3	4	5	5
3,00	2,00	3	4	4	5	3	2	4
5,00	4,00	3	4	4	3	2	5	4
5,00	4,00	5	3	4	5	3	4	4
2,00	2,00	3	2	4	4	3	2	5
4,00	4,00	3	2	4	3	2	3	4
4,00	3,00	4	5	3	4	1	4	4
3,00	1,00	3	2	5	1	1	2	3
4,00	3,00	4	5	1	1	2	5	5
5,00	4,00	5	4	5	4	4	4	5

5,00	5,00	5	5	5	3	1	5	5
3,00	3,00	2	2	3	2	2	4	4
5,00	4,00	5	1	3	4	1	1	5
4,00	3,00	4	5	5	5	3	5	3
3,00	3,00	3	4	2	5	3	5	5
4,00	1,00	3	4	3	4	3	4	3
5,00	1,00	5	1	5	1	1	1	5
4,00	3,00	2	5	4	3	1	5	2
5,00	3,00	5	4	3	5	4	4	5
3,00	2,00	4	3	3	2	2	3	2
4,00	4,00	5	4	4	4	1	3	4
4,00	4,00	5	4	4	3	2	4	3
1,00	3,00	2	1	5	5	5	1	3
5,00	3,00	5	2	5	5	3	3	5
4,00	2,00	3	3	4	3	4	3	4
3,00	5,00	5	4	2	3	5	5	5
5,00	5,00	5	5	5	5	2	5	5
4,00	4,00	4	5	3	4	3	5	5
5,00	2,00	5	3	2	4	2	5	5
4,00	4,00	5	3	5	5	4	4	5
3,00	1,00	4	4	2	4	2	3	5
1,00	3,00	2	1	2	5	5	1	5
2,00	3,00	4	2	4	5	5	1	4
3,00	2,00	1	2	5	1	3	2	1
4,00	5,00	5	1	3	2	1	1	2
5,00	2,00	2	3	1	2	1	3	3
2,00	1,00	5	5	5	1	1	5	2

T1_MOT_2	T1_MOT_2	T1_MOT_2	T1_MOT_2	T1_MOT_2	T1_MOT_2	T1_MOT_3	fact1_T0	fact2_T0
3	1	5	3	5	5	4	20,00	12,00
2	1	4	3	1	5	4	15,00	15,00
5	2	3	4	2	5	1	15,00	13,00
5	5	5	1	5	5	3	25,00	6,00
4	2	3	3	2	5	3	21,00	15,00
5	1	2	5	1	5	2	9,00	15,00
5	1	4	4	1	5	5	8,00	15,00
5	1	1	2	1	5	3	11,00	14,00
5	1	4	5	1	5	5	13,00	15,00
4	1	5	5	1	5	4	14,00	13,00
5	3	5	4	3	5	5	22,00	10,00
3	2	4	3	2	5	3	12,00	7,00
5	2	4	3	1	5	1	16,00	15,00
5	3	3	4	3	5	5	19,00	15,00
5	2	1	3	1	5	4	17,00	12,00
2	3	3	2	2	5	2	20,00	14,00
4	3	1	4	2	5	2	14,00	13,00
4	2	4	2	1	5	2	12,00	12,00
5	2	4	3	2	5	4	12,00	15,00
3	1	4	2	1	3	2	11,00	14,00
4	1	3	4	1	5	5	13,00	14,00
5	1	1	1	1	5	1	10,00	15,00
5	4	5	5	2	5	5	21,00	9,00
4	1	4	2	1	5	2	15,00	14,00
5	3	5	5	2	5	5	15,00	15,00
5	2	4	2	2	3	2	18,00	12,00
1	2	3	3	2	4	2	16,00	6,00
4	5	5	5	5	5	3	15,00	15,00
5	1	2	3	1	5	2	6,00	15,00
3	1	1	5	1	5	2	8,00	15,00
3	3	4	1	3	5	4	7,00	14,00
3	1	5	4	1	4	2	9,00	15,00
5	1	5	4	3	5	5	15,00	15,00
5	4	5	3	3	5	5	16,00	15,00
1	1	1	5	1	5	3	9,00	12,00
5	1	1	3	1	5	3	7,00	10,00
5	3	4	3	3	5	5	17,00	15,00
4	1	2	4	1	5	3	8,00	14,00
5	3	5	5	3	5	3	12,00	13,00
5	2	3	2	1	5	3	7,00	8,00
5	1	3	5	1	4	5	11,00	13,00
4	2	4	3	4	5	3	11,00	15,00
2	1	4	3	1	4	2	8,00	6,00
5	1	5	5	5	5	5	23,00	15,00
4	2	5	4	2	4	3	15,00	15,00
5	1	2	1	1	5	2	10,00	15,00
5	2	4	3	2	5	3	19,00	13,00
5	1	5	5	1	5	5	17,00	15,00
5	4	5	4	3	5	5	17,00	14,00

5	2	1	1	1	3	2	9,00	6,00
4	2	3	3	2	5	3	22,00	15,00
4	1	3	2	2	4	2	10,00	9,00
4	3	2	2	3	4	4	16,00	8,00
5	2	5	3	5	5	4	21,00	15,00
2	1	1	5	1	5	3	6,00	3,00
4	1	4	3	1	5	4	18,00	11,00
5	1	3	4	1	5	3	14,00	14,00
4	3	4	5	3	5	3	20,00	15,00
4	2	3	3	2	5	3	13,00	13,00
2	1	5	5	2	5	3	11,00	5,00
5	1	2	2	1	5	2	8,00	15,00
5	2	5	4	1	5	5	11,00	6,00
1	1	1	1	1	4	2	13,00	9,00
5	1	3	4	1	5	2	12,00	10,00
4	2	4	2	1	3	3	13,00	15,00
5	2	4	3	2	5	3	11,00	14,00
3	1	1	1	1	3	1	7,00	6,00
3	2	4	1	1	2	2	14,00	8,00
5	1	3	2	1	5	5	17,00	13,00
5	5	2	1	4	3	3	13,00	3,00
5	3	4	4	4	5	5	16,00	15,00
5	5	5	4	5	5	5	25,00	14,00
5	3	5	3	2	5	5	14,00	13,00
4	2	3	3	2	5	3	19,00	15,00
4	2	2	2	1	5	3	13,00	12,00
5	5	4	4	4	5	5	21,00	12,00
4	3	3	2	1	3	2	18,00	14,00
2	1	1	5	1	4	3	13,00	15,00
5	2	4	1	3	5	2	17,00	15,00
4	3	2	3	2	5	3	16,00	14,00
5	1	5	1	3	5	5	17,00	15,00
3	2	3	2	1	2	3	12,00	15,00
4	2	5	1	2	5	2	20,00	3,00
5	2	5	5	3	4	5	12,00	13,00
5	1	5	2	2	5	5	20,00	13,00
5	2	5	4	2	5	4	14,00	11,00
5	2	4	5	1	5	5	9,00	3,00
4	3	3	1	3	3	1	12,00	12,00
5	1	1	5	1	5	4	10,00	14,00
5	2	3	3	2	5	5	10,00	9,00
4	2	3	4	1	4	2	19,00	15,00
5	2	5	2	1	4	2	16,00	15,00
5	3	4	5	3	4	4	20,00	10,00
2	2	2	3	2	4	3	15,00	10,00
4	1	3	4	1	3	2	14,00	15,00
5	1	2	2	1	2	2	10,00	11,00
3	1	4	1	1	4	2	14,00	15,00
5	1	5	2	1	4	4	14,00	6,00
2	1	5	1	1	5	3	11,00	13,00

4	1	3	3	1	3	3	8,00	9,00
5	1	2	5	1	4	4	12,00	7,00
5	2	2	2	1	5	2	14,00	15,00
3	1	1	5	1	5	1	5,00	4,00
5	1	1	1	1	4	2	6,00	8,00
5	3	4	3	1	4	4	17,00	7,00
4	2	5	4	1	5	5	18,00	3,00
2	1	5	2	1	2	3	14,00	12,00
5	1	5	3	1	4	4	17,00	14,00
5	2	4	1	1	4	2	10,00	13,00
5	2	1	3	1	5	3	8,00	10,00
5	1	3	4	2	5	5	17,00	7,00
5	2	3	1	3	5	5	16,00	3,00
5	1	5	4	1	5	3	16,00	15,00
4	1	4	1	1	5	2	12,00	9,00
2	1	2	4	1	5	5	9,00	14,00
3	1	2	1	1	4	2	5,00	12,00
5	5	5	2	5	5	5	23,00	10,00
5	3	3	3	3	5	5	20,00	11,00
5	1	2	5	1	5	3	11,00	12,00
5	1	4	2	1	4	3	12,00	15,00
5	2	2	3	1	4	3	7,00	15,00
4	1	4	2	1	5	2	8,00	15,00
5	1	1	3	1	5	5	9,00	15,00
4	2	4	3	1	5	5	13,00	11,00
4	3	3	4	4	4	3	19,00	9,00
5	3	4	4	2	5	4	18,00	12,00
5	1	2	3	1	5	4	6,00	6,00
5	2	4	3	1	5	4	16,00	7,00
5	3	5	4	3	4	5	11,00	8,00
4	1	2	4	1	5	5	11,00	14,00
4	1	3	2	1	5	2	11,00	13,00
4	3	3	3	3	5	3	15,00	15,00
5	4	3	2	1	4	5	10,00	9,00
2	2	5	5	3	3	3	20,00	5,00
4	4	1	3	2	4	3	18,00	6,00
5	2	4	4	2	5	4	13,00	13,00
5	1	1	3	1	5	2	12,00	3,00
2	1	4	2	1	3	1	11,00	6,00
5	1	2	2	5	5	4	14,00	14,00
4	1	3	3	1	4	4	18,00	11,00
2	1	4	4	1	5	1	9,00	11,00
5	3	4	5	2	5	5	14,00	13,00
4	3	2	2	3	4	4	10,00	7,00
3	2	3	3	2	3	3	8,00	14,00
4	3	3	3	2	4	3	16,00	14,00
4	2	3	2	3	5	2	15,00	13,00
1	1	3	1	1	5	1	13,00	15,00
5	2	2	5	3	5	5	19,00	12,00
5	4	1	3	4	5	3	21,00	12,00

4	3	5	4	3	5	4	12,00	15,00
5	5	5	2	5	5	5	24,00	15,00
5	5	5	1	5	5	3	21,00	14,00
5	1	4	3	1	5	3	14,00	15,00
5	5	5	5	5	5	5	23,00	15,00
4	1	3	4	1	4	5	10,00	13,00
5	5	5	4	5	5	2	25,00	15,00
3	5	3	1	1	5	4	16,00	9,00
5	1	4	4	1	5	4	15,00	15,00
5	1	3	1	1	5	3	10,00	12,00
5	1	2	1	1	5	1	15,00	11,00
4	1	3	2	1	5	4	12,00	15,00
3	1	1	2	2	3	3	11,00	12,00
5	3	1	2	3	5	5	16,00	7,00
5	1	4	1	1	5	2	11,00	14,00
5	2	5	3	2	5	3	15,00	15,00
5	2	3	4	2	5	4	18,00	14,00
4	4	5	2	1	5	4	16,00	13,00
4	3	3	3	2	5	4	17,00	15,00
1	1	3	5	2	2	4	16,00	11,00
4	1	3	4	1	5	4	10,00	15,00
5	2	5	5	4	5	5	15,00	12,00
5	1	2	3	1	3	2	8,00	14,00
3	1	1	2	1	4	1	11,00	15,00
5	1	3	1	1	5	2	16,00	14,00
5	2	4	4	3	5	5	19,00	9,00
5	2	2	1	1	5	3	11,00	15,00
5	2	2	2	1	4	5	19,00	12,00
5	1	2	1	1	4	3	8,00	12,00
3	1	4	3	4	4	1	14,00	11,00
4	3	5	2	3	5	5	22,00	11,00
3	2	3	1	1	5	3	8,00	13,00
4	1	1	4	1	3	3	10,00	15,00
2	1	5	2	1	5	5	10,00	15,00
4	1	1	2	1	5	3	5,00	11,00
5	4	5	3	5	5	3	25,00	11,00
4	1	5	4	1	5	4	20,00	15,00
5	4	5	5	1	5	5	19,00	15,00
5	5	5	3	5	5	5	21,00	11,00
5	4	5	5	4	5	5	21,00	14,00
5	1	5	1	1	5	4	9,00	14,00
5	4	4	4	5	5	5	22,00	15,00
5	3	4	1	1	5	1	15,00	14,00
5	2	3	3	4	5	5	16,00	13,00
5	2	3	1	2	5	4	15,00	12,00
5	3	4	4	3	5	4	18,00	15,00
4	2	2	3	3	5	3	13,00	15,00
5	2	2	2	2	5	5	10,00	12,00
4	5	4	4	4	4	4	23,00	14,00
5	1	4	3	1	5	3	12,00	8,00

5	1	3	3	1	5	4	10,00	15,00
5	3	4	4	3	5	3	17,00	14,00
4	1	4	3	1	5	5	12,00	12,00
5	4	4	5	4	4	5	19,00	14,00
5	1	4	2	1	5	4	10,00	15,00
1	1	5	3	1	5	1	19,00	12,00
4	4	3	2	2	5	5	14,00	14,00
5	1	3	2	1	5	4	11,00	11,00
4	2	3	3	1	5	4	16,00	15,00
3	2	2	3	2	5	4	15,00	14,00
3	2	4	2	3	5	4	11,00	15,00
5	2	4	3	2	5	4	13,00	12,00
5	3	4	4	5	5	4	15,00	11,00
5	3	5	4	3	5	4	14,00	15,00
4	3	3	3	2	5	2	18,00	14,00
5	4	4	2	3	4	4	16,00	15,00
1	1	3	2	3	4	4	17,00	11,00
4	4	4	3	3	3	3	18,00	12,00
5	5	5	4	4	5	5	16,00	15,00
4	2	4	3	2	5	3	19,00	10,00
4	2	2	2	1	4	2	10,00	13,00
5	4	5	1	5	5	3	17,00	12,00
4	3	4	1	2	5	5	22,00	9,00
3	1	2	3	1	4	3	12,00	9,00
5	2	4	1	1	5	3	15,00	13,00
5	3	3	1	2	4	4	13,00	15,00
5	3	2	1	4	3	3	16,00	9,00
3	4	3	3	1	4	5	10,00	15,00
4	2	2	2	2	5	2	7,00	6,00
4	2	4	3	2	5	4	20,00	15,00
3	2	3	5	2	2	3	15,00	12,00
5	4	4	4	3	5	4	14,00	15,00
5	2	3	4	2	5	3	19,00	14,00
5	5	5	5	5	5	5	24,00	14,00
5	4	4	3	3	5	5	19,00	9,00
5	1	3	2	1	5	1	16,00	14,00
5	1	2	1	1	4	3	10,00	10,00
4	3	3	2	3	3	2	13,00	9,00
4	3	2	2	2	4	3	14,00	11,00
5	3	5	2	5	5	1	19,00	13,00
4	2	3	3	4	5	5	18,00	14,00
2	2	5	1	1	4	3	13,00	13,00
5	2	2	3	2	5	3	14,00	14,00
5	3	4	1	1	5	2	13,00	15,00
4	2	4	4	2	1	1	16,00	6,00
4	2	3	3	2	5	5	17,00	13,00
3	2	4	2	2	5	4	11,00	12,00
4	1	2	1	1	5	5	14,00	9,00
4	1	3	2	1	5	4	16,00	9,00
5	2	4	3	3	5	4	11,00	12,00

5	2	5	4	1	5	5	11,00	11,00
3	1	2	3	2	3	3	15,00	4,00
5	1	5	4	1	5	3	9,00	3,00
5	2	5	4	1	5	4	18,00	15,00
4	1	4	2	1	5	3	15,00	15,00
3	2	2	3	4	5	3	11,00	14,00
3	1	5	1	1	1	5	5,00	3,00
5	1	4	1	1	5	4	10,00	15,00
5	4	5	3	4	5	3	17,00	11,00
4	2	2	2	2	3	2	13,00	12,00
4	1	3	4	1	5	5	15,00	12,00
4	2	4	4	1	5	3	9,00	11,00
2	1	4	1	1	4	2	20,00	7,00
4	4	5	3	1	5	4	20,00	9,00
5	5	4	3	5	5	3	20,00	11,00
5	5	3	1	5	5	2	20,00	12,00
4	1	5	5	1	5	5	15,00	13,00
5	2	4	5	4	4	4	16,00	14,00
5	3	4	4	2	5	3	21,00	11,00
4	1	5	4	2	5	5	18,00	10,00
4	1	4	3	1	5	2	9,00	15,00
1	5	5	4	5	3	1	25,00	7,00
3	2	5	1	4	5	5	19,00	3,00
4	5	1	1	1	5	2	13,00	10,00
2	4	1	1	1	5	4	8,00	8,00
4	2	5	3	2	5	3	11,00	11,00
1	1	1	3	1	5	1	6,00	15,00

fact3_T0	fact4_T0	fact5_T0	fact6_T0	fact7_T0	fact8_T0	fact1_T1	fact2_T1	fact3_T1
12,00	22,00	23,00	15,00	17,00	7,00	17,00	13,00	16,00
10,00	20,00	20,00	9,00	17,00	6,00	13,00	15,00	14,00
10,00	15,00	25,00	15,00	15,00	6,00	14,00	10,00	11,00
17,00	14,00	17,00	14,00	14,00	9,00	25,00	3,00	20,00
17,00	25,00	25,00	15,00	20,00	10,00	13,00	12,00	12,00
7,00	23,00	22,00	7,00	17,00	8,00	7,00	15,00	13,00
6,00	24,00	23,00	15,00	19,00	9,00	12,00	15,00	17,00
7,00	15,00	19,00	12,00	14,00	7,00	9,00	9,00	7,00
11,00	19,00	24,00	14,00	20,00	9,00	12,00	15,00	15,00
10,00	18,00	22,00	15,00	18,00	10,00	11,00	15,00	11,00
18,00	22,00	21,00	15,00	16,00	8,00	18,00	12,00	16,00
12,00	21,00	17,00	12,00	16,00	8,00	13,00	9,00	14,00
17,00	20,00	20,00	15,00	20,00	9,00	15,00	15,00	16,00
17,00	24,00	23,00	12,00	19,00	9,00	16,00	15,00	13,00
15,00	20,00	17,00	13,00	18,00	9,00	10,00	14,00	5,00
16,00	15,00	16,00	8,00	17,00	9,00	16,00	10,00	14,00
10,00	18,00	17,00	12,00	17,00	6,00	12,00	13,00	6,00
11,00	14,00	16,00	13,00	15,00	8,00	13,00	12,00	12,00
11,00	18,00	23,00	15,00	19,00	7,00	16,00	15,00	12,00
13,00	12,00	23,00	13,00	16,00	5,00	10,00	13,00	10,00
9,00	21,00	20,00	10,00	17,00	10,00	11,00	15,00	9,00
12,00	25,00	21,00	14,00	19,00	10,00	8,00	15,00	9,00
17,00	19,00	23,00	13,00	18,00	8,00	21,00	15,00	18,00
15,00	19,00	21,00	11,00	15,00	9,00	11,00	12,00	16,00
16,00	20,00	23,00	15,00	15,00	5,00	17,00	15,00	18,00
15,00	18,00	21,00	14,00	19,00	7,00	13,00	8,00	15,00
13,00	15,00	16,00	9,00	12,00	6,00	10,00	5,00	8,00
9,00	12,00	22,00	10,00	11,00	3,00	23,00	14,00	13,00
6,00	9,00	18,00	9,00	12,00	2,00	7,00	13,00	7,00
4,00	13,00	17,00	8,00	16,00	10,00	9,00	15,00	5,00
11,00	20,00	19,00	14,00	18,00	10,00	13,00	15,00	10,00
11,00	18,00	18,00	13,00	15,00	7,00	11,00	13,00	11,00
14,00	17,00	21,00	13,00	19,00	10,00	13,00	15,00	12,00
15,00	15,00	24,00	15,00	17,00	8,00	19,00	15,00	16,00
4,00	12,00	19,00	10,00	9,00	6,00	7,00	14,00	4,00
4,00	9,00	20,00	14,00	14,00	7,00	7,00	15,00	5,00
15,00	19,00	22,00	13,00	18,00	6,00	18,00	15,00	17,00
8,00	19,00	16,00	13,00	18,00	7,00	7,00	14,00	6,00
11,00	9,00	22,00	11,00	11,00	7,00	17,00	15,00	17,00
12,00	9,00	18,00	13,00	6,00	6,00	9,00	8,00	8,00
9,00	16,00	23,00	13,00	13,00	8,00	13,00	11,00	15,00
13,00	18,00	23,00	12,00	15,00	10,00	19,00	15,00	10,00
7,00	13,00	21,00	8,00	13,00	5,00	8,00	6,00	9,00
17,00	20,00	22,00	14,00	20,00	10,00	17,00	15,00	12,00
15,00	23,00	25,00	15,00	20,00	9,00	15,00	15,00	16,00
11,00	11,00	22,00	15,00	17,00	9,00	7,00	12,00	7,00
18,00	23,00	24,00	15,00	19,00	6,00	15,00	13,00	16,00
12,00	24,00	25,00	15,00	20,00	8,00	12,00	15,00	13,00
15,00	14,00	23,00	14,00	20,00	10,00	21,00	15,00	20,00

6,00	12,00	18,00	14,00	10,00	4,00	10,00	8,00	7,00
19,00	19,00	17,00	14,00	17,00	3,00	13,00	12,00	12,00
10,00	16,00	21,00	12,00	14,00	7,00	13,00	12,00	9,00
13,00	20,00	22,00	15,00	18,00	9,00	17,00	7,00	9,00
15,00	19,00	22,00	13,00	19,00	10,00	20,00	12,00	14,00
4,00	10,00	17,00	3,00	10,00	10,00	6,00	3,00	4,00
13,00	13,00	15,00	14,00	16,00	10,00	10,00	8,00	13,00
14,00	18,00	22,00	14,00	13,00	10,00	12,00	15,00	14,00
18,00	18,00	24,00	15,00	17,00	9,00	17,00	15,00	15,00
12,00	15,00	22,00	13,00	15,00	6,00	13,00	13,00	10,00
12,00	10,00	22,00	9,00	15,00	10,00	13,00	8,00	12,00
5,00	12,00	22,00	15,00	16,00	9,00	7,00	15,00	5,00
11,00	7,00	22,00	15,00	17,00	5,00	14,00	9,00	15,00
11,00	17,00	16,00	9,00	15,00	8,00	11,00	4,00	8,00
16,00	12,00	19,00	15,00	18,00	7,00	12,00	11,00	15,00
13,00	15,00	17,00	12,00	15,00	8,00	10,00	15,00	12,00
10,00	20,00	23,00	15,00	18,00	7,00	14,00	15,00	12,00
5,00	11,00	9,00	8,00	6,00	4,00	7,00	4,00	4,00
12,00	10,00	20,00	11,00	5,00	3,00	13,00	6,00	14,00
12,00	18,00	20,00	15,00	18,00	6,00	16,00	13,00	12,00
11,00	10,00	17,00	14,00	17,00	6,00	19,00	3,00	10,00
19,00	20,00	23,00	13,00	16,00	10,00	19,00	14,00	16,00
20,00	25,00	25,00	15,00	20,00	6,00	25,00	14,00	20,00
15,00	18,00	22,00	15,00	19,00	8,00	15,00	12,00	12,00
19,00	21,00	24,00	13,00	14,00	9,00	13,00	12,00	12,00
7,00	14,00	18,00	13,00	16,00	10,00	11,00	8,00	11,00
18,00	18,00	25,00	14,00	18,00	10,00	22,00	14,00	16,00
15,00	23,00	22,00	13,00	18,00	6,00	14,00	11,00	11,00
15,00	14,00	21,00	12,00	13,00	8,00	12,00	15,00	11,00
13,00	9,00	20,00	14,00	16,00	10,00	17,00	15,00	11,00
13,00	19,00	20,00	15,00	16,00	2,00	14,00	9,00	7,00
18,00	11,00	24,00	14,00	15,00	6,00	19,00	15,00	19,00
12,00	10,00	19,00	13,00	10,00	8,00	14,00	15,00	11,00
16,00	14,00	25,00	15,00	16,00	10,00	13,00	5,00	17,00
17,00	10,00	22,00	12,00	11,00	8,00	18,00	15,00	16,00
16,00	18,00	24,00	14,00	17,00	8,00	14,00	13,00	17,00
17,00	15,00	25,00	14,00	16,00	10,00	15,00	9,00	15,00
8,00	12,00	22,00	14,00	12,00	2,00	13,00	6,00	11,00
10,00	8,00	20,00	13,00	8,00	6,00	17,00	15,00	11,00
6,00	15,00	22,00	14,00	16,00	7,00	8,00	13,00	8,00
8,00	19,00	22,00	14,00	20,00	9,00	14,00	12,00	9,00
15,00	20,00	24,00	12,00	17,00	7,00	13,00	14,00	13,00
20,00	20,00	24,00	15,00	16,00	2,00	11,00	15,00	19,00
18,00	20,00	25,00	13,00	17,00	9,00	15,00	15,00	14,00
8,00	13,00	19,00	6,00	12,00	4,00	14,00	9,00	10,00
15,00	22,00	21,00	15,00	19,00	7,00	8,00	11,00	9,00
6,00	12,00	19,00	11,00	12,00	3,00	7,00	7,00	5,00
14,00	16,00	25,00	12,00	15,00	5,00	11,00	15,00	12,00
17,00	7,00	22,00	14,00	16,00	8,00	12,00	5,00	14,00
10,00	8,00	14,00	10,00	14,00	4,00	11,00	15,00	15,00

9,00	6,00	19,00	14,00	11,00	5,00	10,00	14,00	10,00
11,00	15,00	24,00	15,00	10,00	9,00	9,00	10,00	8,00
13,00	16,00	18,00	14,00	17,00	4,00	15,00	15,00	11,00
4,00	10,00	14,00	8,00	8,00	2,00	5,00	7,00	4,00
4,00	14,00	16,00	11,00	13,00	7,00	8,00	7,00	4,00
18,00	20,00	19,00	15,00	16,00	9,00	16,00	10,00	17,00
17,00	12,00	22,00	12,00	13,00	10,00	15,00	3,00	19,00
19,00	7,00	17,00	13,00	15,00	8,00	13,00	12,00	17,00
20,00	12,00	20,00	14,00	18,00	9,00	16,00	9,00	18,00
9,00	8,00	16,00	15,00	15,00	3,00	14,00	12,00	13,00
10,00	16,00	14,00	7,00	12,00	7,00	8,00	12,00	5,00
14,00	16,00	21,00	14,00	18,00	5,00	12,00	10,00	11,00
12,00	12,00	21,00	15,00	19,00	10,00	16,00	11,00	15,00
20,00	14,00	24,00	14,00	19,00	8,00	9,00	15,00	13,00
14,00	12,00	20,00	12,00	17,00	9,00	12,00	11,00	13,00
4,00	18,00	25,00	11,00	20,00	6,00	8,00	14,00	5,00
7,00	9,00	14,00	8,00	14,00	8,00	6,00	10,00	7,00
18,00	12,00	16,00	11,00	16,00	8,00	25,00	12,00	20,00
18,00	19,00	22,00	15,00	17,00	6,00	21,00	15,00	16,00
10,00	20,00	16,00	12,00	14,00	9,00	10,00	15,00	8,00
11,00	14,00	18,00	14,00	18,00	8,00	10,00	11,00	11,00
7,00	10,00	19,00	10,00	11,00	4,00	8,00	15,00	9,00
9,00	15,00	20,00	13,00	14,00	8,00	8,00	15,00	11,00
7,00	14,00	23,00	15,00	17,00	8,00	7,00	12,00	4,00
15,00	20,00	18,00	11,00	18,00	5,00	14,00	10,00	12,00
10,00	17,00	13,00	6,00	15,00	6,00	19,00	11,00	13,00
20,00	20,00	21,00	14,00	18,00	6,00	19,00	12,00	18,00
6,00	16,00	10,00	14,00	11,00	6,00	10,00	11,00	9,00
15,00	19,00	24,00	15,00	18,00	10,00	14,00	5,00	15,00
12,00	12,00	19,00	13,00	15,00	5,00	17,00	12,00	14,00
13,00	21,00	24,00	13,00	17,00	10,00	10,00	11,00	9,00
12,00	10,00	17,00	12,00	12,00	8,00	12,00	13,00	11,00
13,00	20,00	24,00	11,00	16,00	8,00	15,00	14,00	13,00
10,00	14,00	18,00	15,00	14,00	7,00	12,00	12,00	9,00
20,00	13,00	23,00	8,00	9,00	10,00	18,00	7,00	20,00
13,00	20,00	18,00	8,00	14,00	7,00	14,00	7,00	8,00
12,00	15,00	19,00	12,00	18,00	5,00	15,00	15,00	15,00
4,00	13,00	14,00	12,00	12,00	10,00	11,00	11,00	4,00
17,00	11,00	20,00	9,00	10,00	5,00	9,00	8,00	13,00
15,00	23,00	22,00	13,00	20,00	8,00	18,00	13,00	12,00
11,00	19,00	14,00	10,00	16,00	6,00	9,00	13,00	9,00
12,00	12,00	21,00	15,00	9,00	4,00	17,00	12,00	13,00
15,00	25,00	21,00	13,00	20,00	9,00	15,00	15,00	11,00
16,00	16,00	10,00	10,00	10,00	8,00	17,00	12,00	15,00
8,00	17,00	15,00	10,00	16,00	7,00	10,00	15,00	9,00
17,00	17,00	21,00	10,00	16,00	6,00	15,00	12,00	14,00
10,00	12,00	19,00	9,00	14,00	4,00	16,00	13,00	15,00
12,00	12,00	24,00	13,00	12,00	8,00	10,00	15,00	10,00
19,00	21,00	21,00	15,00	18,00	9,00	16,00	5,00	14,00
20,00	20,00	19,00	15,00	18,00	5,00	17,00	9,00	12,00

13,00	16,00	23,00	10,00	14,00	2,00	17,00	14,00	16,00
13,00	20,00	20,00	15,00	11,00	10,00	25,00	15,00	18,00
18,00	14,00	21,00	15,00	19,00	3,00	25,00	12,00	19,00
10,00	14,00	17,00	13,00	15,00	9,00	15,00	13,00	15,00
19,00	25,00	23,00	14,00	20,00	9,00	24,00	15,00	19,00
11,00	16,00	22,00	9,00	14,00	3,00	9,00	15,00	9,00
17,00	19,00	24,00	15,00	18,00	10,00	25,00	15,00	18,00
9,00	23,00	13,00	7,00	10,00	6,00	15,00	13,00	11,00
16,00	15,00	23,00	15,00	19,00	9,00	14,00	15,00	19,00
11,00	11,00	20,00	15,00	14,00	10,00	9,00	10,00	10,00
9,00	17,00	17,00	14,00	12,00	9,00	15,00	14,00	10,00
14,00	21,00	25,00	15,00	19,00	10,00	14,00	15,00	15,00
7,00	10,00	21,00	11,00	17,00	2,00	14,00	11,00	7,00
16,00	22,00	15,00	11,00	12,00	7,00	18,00	9,00	13,00
10,00	11,00	22,00	14,00	11,00	8,00	10,00	15,00	12,00
16,00	21,00	21,00	14,00	19,00	10,00	12,00	14,00	14,00
11,00	23,00	14,00	12,00	17,00	8,00	17,00	14,00	14,00
11,00	15,00	20,00	11,00	17,00	2,00	17,00	12,00	19,00
11,00	17,00	16,00	10,00	15,00	7,00	17,00	13,00	13,00
17,00	14,00	21,00	6,00	15,00	6,00	15,00	13,00	15,00
10,00	18,00	19,00	12,00	16,00	2,00	8,00	15,00	6,00
14,00	22,00	24,00	15,00	20,00	8,00	16,00	12,00	12,00
8,00	19,00	16,00	12,00	13,00	7,00	8,00	8,00	5,00
9,00	13,00	14,00	7,00	9,00	4,00	9,00	10,00	8,00
12,00	14,00	17,00	12,00	14,00	7,00	9,00	13,00	7,00
15,00	20,00	20,00	11,00	17,00	7,00	17,00	9,00	15,00
15,00	9,00	21,00	13,00	14,00	7,00	14,00	9,00	13,00
16,00	24,00	18,00	11,00	14,00	7,00	16,00	13,00	15,00
6,00	18,00	16,00	15,00	15,00	4,00	7,00	12,00	6,00
12,00	14,00	18,00	13,00	12,00	4,00	14,00	8,00	17,00
20,00	19,00	23,00	15,00	18,00	8,00	19,00	14,00	19,00
9,00	10,00	19,00	14,00	17,00	10,00	9,00	12,00	9,00
11,00	9,00	21,00	12,00	19,00	9,00	11,00	11,00	11,00
7,00	23,00	19,00	11,00	19,00	8,00	13,00	15,00	17,00
4,00	10,00	17,00	12,00	11,00	5,00	9,00	11,00	6,00
20,00	23,00	24,00	12,00	16,00	6,00	24,00	14,00	20,00
18,00	21,00	25,00	14,00	17,00	9,00	14,00	15,00	17,00
19,00	22,00	25,00	15,00	17,00	9,00	18,00	14,00	19,00
19,00	23,00	23,00	14,00	20,00	6,00	24,00	14,00	20,00
19,00	19,00	22,00	15,00	20,00	7,00	22,00	15,00	20,00
12,00	17,00	19,00	15,00	17,00	9,00	14,00	15,00	12,00
17,00	24,00	22,00	15,00	18,00	8,00	23,00	15,00	19,00
13,00	17,00	23,00	15,00	15,00	8,00	13,00	11,00	11,00
12,00	18,00	20,00	12,00	16,00	6,00	18,00	14,00	16,00
15,00	18,00	16,00	8,00	16,00	7,00	14,00	13,00	14,00
13,00	18,00	21,00	14,00	18,00	6,00	19,00	14,00	15,00
14,00	20,00	19,00	9,00	16,00	7,00	16,00	12,00	10,00
12,00	21,00	22,00	14,00	17,00	8,00	16,00	14,00	13,00
18,00	23,00	24,00	14,00	15,00	6,00	23,00	12,00	17,00
9,00	21,00	24,00	15,00	18,00	5,00	12,00	9,00	12,00

11,00	22,00	23,00	15,00	15,00	7,00	11,00	12,00	14,00
13,00	20,00	22,00	11,00	15,00	8,00	18,00	13,00	16,00
16,00	7,00	19,00	12,00	17,00	10,00	13,00	12,00	16,00
15,00	19,00	23,00	14,00	17,00	7,00	20,00	15,00	16,00
13,00	20,00	22,00	13,00	17,00	8,00	13,00	13,00	11,00
13,00	11,00	21,00	7,00	16,00	10,00	17,00	11,00	12,00
16,00	21,00	22,00	15,00	20,00	6,00	20,00	14,00	15,00
5,00	10,00	21,00	15,00	13,00	4,00	12,00	10,00	11,00
20,00	20,00	22,00	13,00	12,00	6,00	14,00	14,00	17,00
19,00	20,00	22,00	13,00	13,00	8,00	13,00	11,00	14,00
8,00	21,00	21,00	7,00	15,00	3,00	15,00	15,00	13,00
12,00	17,00	20,00	15,00	19,00	7,00	14,00	11,00	10,00
17,00	18,00	21,00	12,00	19,00	7,00	22,00	15,00	17,00
20,00	20,00	22,00	12,00	13,00	6,00	18,00	11,00	19,00
17,00	20,00	19,00	13,00	15,00	10,00	18,00	13,00	15,00
9,00	21,00	21,00	13,00	18,00	3,00	17,00	13,00	14,00
14,00	15,00	20,00	6,00	17,00	8,00	11,00	8,00	10,00
17,00	19,00	18,00	11,00	10,00	9,00	17,00	13,00	15,00
17,00	19,00	20,00	12,00	17,00	6,00	21,00	15,00	18,00
18,00	13,00	19,00	12,00	16,00	6,00	17,00	12,00	18,00
11,00	13,00	18,00	11,00	13,00	5,00	8,00	8,00	13,00
20,00	18,00	18,00	15,00	17,00	5,00	24,00	10,00	20,00
18,00	21,00	21,00	10,00	17,00	10,00	15,00	8,00	15,00
8,00	17,00	13,00	12,00	13,00	6,00	10,00	9,00	6,00
14,00	21,00	19,00	13,00	11,00	8,00	18,00	11,00	19,00
16,00	16,00	22,00	13,00	20,00	8,00	18,00	14,00	15,00
14,00	21,00	17,00	13,00	13,00	9,00	17,00	9,00	8,00
8,00	16,00	22,00	9,00	19,00	2,00	16,00	14,00	14,00
6,00	7,00	14,00	12,00	10,00	2,00	11,00	11,00	8,00
19,00	20,00	24,00	11,00	18,00	8,00	16,00	14,00	16,00
12,00	12,00	18,00	10,00	13,00	4,00	10,00	14,00	8,00
15,00	22,00	20,00	13,00	16,00	8,00	20,00	12,00	18,00
17,00	16,00	23,00	13,00	15,00	7,00	14,00	13,00	16,00
20,00	24,00	25,00	15,00	20,00	10,00	25,00	11,00	18,00
16,00	17,00	18,00	15,00	15,00	8,00	20,00	14,00	17,00
20,00	12,00	21,00	15,00	9,00	10,00	12,00	12,00	18,00
12,00	19,00	15,00	14,00	11,00	10,00	8,00	9,00	8,00
9,00	15,00	20,00	11,00	11,00	9,00	14,00	7,00	10,00
10,00	18,00	16,00	11,00	14,00	6,00	14,00	9,00	10,00
15,00	18,00	24,00	15,00	13,00	10,00	18,00	12,00	11,00
16,00	19,00	19,00	13,00	19,00	10,00	17,00	15,00	12,00
18,00	15,00	18,00	8,00	15,00	8,00	15,00	10,00	18,00
12,00	22,00	20,00	14,00	17,00	8,00	13,00	14,00	10,00
8,00	17,00	16,00	15,00	16,00	6,00	17,00	12,00	17,00
19,00	14,00	20,00	11,00	9,00	9,00	15,00	5,00	18,00
9,00	16,00	23,00	13,00	19,00	8,00	14,00	8,00	10,00
8,00	14,00	20,00	13,00	17,00	10,00	13,00	14,00	12,00
9,00	17,00	19,00	11,00	12,00	6,00	9,00	6,00	5,00
10,00	16,00	23,00	15,00	17,00	3,00	9,00	14,00	6,00
10,00	16,00	19,00	14,00	12,00	9,00	16,00	12,00	15,00

11,00	21,00	23,00	11,00	19,00	10,00	11,00	15,00	13,00
11,00	15,00	13,00	14,00	12,00	5,00	11,00	8,00	9,00
8,00	12,00	19,00	15,00	13,00	10,00	11,00	3,00	13,00
14,00	22,00	19,00	14,00	18,00	9,00	16,00	15,00	17,00
15,00	18,00	21,00	14,00	14,00	6,00	15,00	13,00	18,00
12,00	20,00	20,00	12,00	13,00	5,00	16,00	11,00	11,00
8,00	5,00	13,00	15,00	16,00	10,00	5,00	3,00	8,00
10,00	16,00	20,00	15,00	18,00	9,00	11,00	15,00	11,00
16,00	16,00	21,00	13,00	14,00	6,00	22,00	12,00	18,00
13,00	18,00	12,00	13,00	16,00	9,00	11,00	9,00	10,00
14,00	21,00	19,00	13,00	19,00	9,00	11,00	11,00	14,00
12,00	18,00	15,00	11,00	15,00	8,00	12,00	12,00	13,00
19,00	12,00	17,00	7,00	12,00	9,00	16,00	3,00	19,00
20,00	17,00	20,00	13,00	17,00	8,00	18,00	6,00	17,00
13,00	21,00	23,00	14,00	12,00	8,00	22,00	9,00	17,00
9,00	12,00	15,00	15,00	15,00	6,00	22,00	13,00	16,00
20,00	21,00	25,00	15,00	20,00	8,00	14,00	14,00	18,00
16,00	20,00	25,00	15,00	15,00	8,00	18,00	14,00	17,00
16,00	21,00	22,00	15,00	19,00	8,00	15,00	12,00	12,00
18,00	20,00	22,00	13,00	20,00	10,00	17,00	10,00	20,00
11,00	11,00	24,00	11,00	11,00	9,00	11,00	11,00	13,00
20,00	19,00	19,00	7,00	7,00	2,00	25,00	3,00	20,00
9,00	17,00	17,00	9,00	18,00	4,00	21,00	5,00	18,00
11,00	19,00	10,00	11,00	11,00	6,00	14,00	7,00	5,00
8,00	12,00	17,00	14,00	19,00	9,00	13,00	4,00	7,00
11,00	10,00	17,00	15,00	10,00	10,00	10,00	9,00	10,00
4,00	8,00	10,00	6,00	17,00	8,00	8,00	15,00	6,00

fact4_T1	fact5_T1	fact6_T1	fact7_T1	fact8_T1
23,00	21,00	11,00	19,00	6,00
21,00	17,00	7,00	18,00	5,00
14,00	24,00	15,00	13,00	10,00
19,00	16,00	15,00	15,00	10,00
17,00	19,00	12,00	15,00	8,00
24,00	25,00	15,00	15,00	7,00
23,00	22,00	13,00	20,00	10,00
10,00	17,00	14,00	13,00	9,00
22,00	25,00	15,00	19,00	10,00
18,00	23,00	13,00	15,00	7,00
24,00	24,00	15,00	19,00	10,00
18,00	22,00	12,00	16,00	9,00
23,00	19,00	15,00	16,00	10,00
23,00	24,00	15,00	20,00	8,00
22,00	23,00	15,00	19,00	9,00
15,00	19,00	6,00	16,00	5,00
12,00	20,00	12,00	17,00	7,00
19,00	18,00	11,00	16,00	8,00
20,00	23,00	13,00	16,00	7,00
12,00	16,00	12,00	12,00	6,00
21,00	22,00	14,00	20,00	10,00
25,00	19,00	14,00	16,00	7,00
25,00	25,00	15,00	20,00	10,00
17,00	22,00	12,00	11,00	5,00
23,00	24,00	15,00	16,00	4,00
12,00	17,00	14,00	12,00	3,00
18,00	14,00	3,00	10,00	9,00
15,00	23,00	11,00	14,00	5,00
14,00	19,00	14,00	14,00	5,00
14,00	24,00	11,00	16,00	6,00
22,00	17,00	9,00	15,00	10,00
12,00	16,00	11,00	16,00	10,00
23,00	22,00	14,00	20,00	10,00
12,00	23,00	12,00	20,00	8,00
13,00	15,00	5,00	13,00	4,00
14,00	19,00	15,00	16,00	7,00
20,00	22,00	15,00	19,00	10,00
20,00	20,00	13,00	15,00	6,00
10,00	25,00	15,00	12,00	9,00
11,00	21,00	15,00	11,00	5,00
17,00	24,00	15,00	14,00	7,00
16,00	17,00	9,00	15,00	10,00
9,00	17,00	6,00	11,00	6,00
19,00	25,00	14,00	20,00	10,00
16,00	21,00	14,00	16,00	8,00
12,00	19,00	15,00	14,00	7,00
21,00	23,00	15,00	17,00	9,00
23,00	25,00	15,00	20,00	9,00
21,00	23,00	15,00	19,00	10,00

10,00	18,00	13,00	10,00	4,00
17,00	19,00	12,00	15,00	8,00
17,00	18,00	11,00	13,00	7,00
18,00	19,00	14,00	16,00	7,00
23,00	21,00	11,00	18,00	10,00
13,00	17,00	5,00	15,00	10,00
16,00	17,00	12,00	18,00	10,00
16,00	22,00	13,00	16,00	10,00
21,00	22,00	10,00	16,00	8,00
14,00	20,00	12,00	16,00	8,00
13,00	25,00	7,00	16,00	10,00
14,00	22,00	15,00	12,00	9,00
19,00	22,00	14,00	18,00	10,00
18,00	10,00	7,00	16,00	10,00
18,00	19,00	14,00	14,00	7,00
15,00	18,00	12,00	12,00	10,00
20,00	20,00	15,00	17,00	7,00
7,00	6,00	7,00	7,00	2,00
6,00	17,00	7,00	6,00	6,00
20,00	20,00	13,00	17,00	7,00
13,00	13,00	14,00	12,00	9,00
18,00	23,00	13,00	19,00	10,00
24,00	24,00	14,00	20,00	10,00
19,00	23,00	15,00	20,00	8,00
17,00	19,00	12,00	15,00	8,00
16,00	16,00	11,00	13,00	9,00
22,00	20,00	14,00	18,00	9,00
20,00	16,00	12,00	13,00	10,00
17,00	22,00	8,00	13,00	8,00
17,00	20,00	15,00	16,00	6,00
16,00	14,00	12,00	14,00	4,00
21,00	21,00	15,00	20,00	9,00
11,00	19,00	11,00	12,00	10,00
12,00	21,00	13,00	14,00	10,00
12,00	25,00	13,00	17,00	5,00
19,00	21,00	15,00	19,00	10,00
21,00	24,00	12,00	19,00	8,00
17,00	24,00	15,00	20,00	3,00
6,00	20,00	14,00	7,00	6,00
16,00	23,00	15,00	18,00	10,00
22,00	22,00	15,00	18,00	9,00
15,00	20,00	11,00	13,00	7,00
12,00	19,00	15,00	13,00	4,00
21,00	24,00	12,00	15,00	10,00
14,00	17,00	6,00	14,00	7,00
12,00	17,00	10,00	10,00	7,00
10,00	14,00	13,00	7,00	4,00
19,00	18,00	11,00	12,00	7,00
7,00	22,00	15,00	15,00	9,00
6,00	16,00	8,00	11,00	3,00

8,00	21,00	12,00	14,00	7,00
12,00	22,00	14,00	13,00	10,00
16,00	19,00	15,00	16,00	4,00
15,00	20,00	10,00	12,00	6,00
11,00	13,00	13,00	12,00	6,00
23,00	18,00	15,00	15,00	9,00
14,00	20,00	13,00	16,00	10,00
6,00	15,00	7,00	10,00	8,00
14,00	19,00	14,00	17,00	10,00
14,00	19,00	14,00	11,00	4,00
24,00	17,00	11,00	16,00	2,00
13,00	23,00	14,00	18,00	5,00
23,00	17,00	15,00	20,00	10,00
13,00	22,00	15,00	14,00	3,00
11,00	15,00	11,00	12,00	9,00
17,00	20,00	8,00	17,00	6,00
12,00	13,00	10,00	13,00	5,00
19,00	22,00	14,00	18,00	5,00
19,00	23,00	15,00	19,00	10,00
21,00	23,00	15,00	17,00	10,00
12,00	17,00	14,00	12,00	6,00
13,00	22,00	15,00	14,00	6,00
13,00	16,00	11,00	12,00	7,00
10,00	22,00	15,00	19,00	10,00
17,00	21,00	14,00	18,00	10,00
16,00	22,00	10,00	14,00	8,00
16,00	22,00	14,00	16,00	6,00
23,00	19,00	14,00	16,00	8,00
16,00	23,00	15,00	18,00	10,00
21,00	21,00	13,00	17,00	6,00
21,00	24,00	14,00	19,00	10,00
13,00	18,00	11,00	11,00	9,00
17,00	22,00	13,00	14,00	9,00
16,00	19,00	14,00	17,00	8,00
15,00	22,00	7,00	9,00	7,00
17,00	15,00	9,00	12,00	6,00
16,00	24,00	13,00	17,00	9,00
22,00	19,00	13,00	16,00	10,00
9,00	18,00	8,00	7,00	9,00
20,00	19,00	14,00	18,00	3,00
20,00	18,00	10,00	15,00	7,00
17,00	20,00	9,00	9,00	7,00
24,00	25,00	14,00	20,00	7,00
16,00	16,00	12,00	18,00	5,00
14,00	19,00	11,00	12,00	7,00
17,00	17,00	10,00	14,00	8,00
15,00	20,00	12,00	14,00	5,00
12,00	15,00	7,00	10,00	6,00
14,00	20,00	14,00	17,00	10,00
21,00	22,00	15,00	16,00	6,00

17,00	20,00	12,00	15,00	3,00
19,00	22,00	15,00	19,00	10,00
15,00	20,00	13,00	13,00	3,00
17,00	21,00	14,00	14,00	7,00
25,00	24,00	15,00	20,00	9,00
15,00	18,00	12,00	14,00	4,00
24,00	24,00	15,00	17,00	10,00
23,00	10,00	8,00	15,00	4,00
23,00	24,00	15,00	18,00	10,00
10,00	18,00	13,00	13,00	9,00
17,00	19,00	15,00	15,00	8,00
21,00	20,00	13,00	18,00	10,00
12,00	17,00	11,00	11,00	3,00
21,00	15,00	13,00	15,00	6,00
15,00	20,00	15,00	14,00	8,00
17,00	19,00	13,00	16,00	7,00
23,00	21,00	14,00	18,00	8,00
21,00	21,00	10,00	14,00	5,00
18,00	23,00	13,00	16,00	6,00
20,00	25,00	5,00	11,00	3,00
14,00	20,00	12,00	18,00	3,00
18,00	25,00	15,00	20,00	7,00
11,00	20,00	14,00	12,00	5,00
14,00	14,00	8,00	8,00	2,00
16,00	17,00	14,00	15,00	7,00
16,00	18,00	13,00	17,00	9,00
14,00	20,00	15,00	18,00	6,00
19,00	18,00	10,00	15,00	9,00
12,00	18,00	15,00	12,00	2,00
14,00	17,00	9,00	12,00	4,00
19,00	19,00	14,00	18,00	7,00
12,00	19,00	12,00	13,00	9,00
13,00	24,00	14,00	10,00	7,00
19,00	15,00	10,00	14,00	8,00
13,00	19,00	13,00	15,00	7,00
22,00	20,00	14,00	17,00	5,00
20,00	24,00	13,00	17,00	6,00
21,00	24,00	15,00	18,00	3,00
25,00	22,00	15,00	19,00	7,00
20,00	25,00	14,00	19,00	9,00
15,00	19,00	15,00	18,00	10,00
24,00	23,00	15,00	19,00	9,00
12,00	21,00	15,00	14,00	6,00
16,00	23,00	14,00	17,00	7,00
16,00	18,00	12,00	17,00	8,00
16,00	23,00	14,00	18,00	7,00
10,00	19,00	11,00	12,00	5,00
21,00	20,00	15,00	20,00	8,00
21,00	21,00	12,00	16,00	8,00
21,00	21,00	14,00	16,00	6,00

19,00	22,00	15,00	19,00	10,00
19,00	22,00	13,00	17,00	8,00
10,00	21,00	13,00	17,00	8,00
16,00	24,00	14,00	18,00	7,00
22,00	19,00	14,00	17,00	6,00
14,00	20,00	7,00	16,00	10,00
22,00	20,00	11,00	18,00	4,00
15,00	20,00	15,00	18,00	7,00
17,00	21,00	11,00	16,00	6,00
15,00	20,00	10,00	18,00	9,00
19,00	17,00	10,00	17,00	7,00
16,00	19,00	15,00	15,00	5,00
21,00	22,00	15,00	18,00	7,00
21,00	24,00	15,00	17,00	10,00
24,00	22,00	12,00	16,00	5,00
22,00	19,00	14,00	17,00	7,00
18,00	11,00	7,00	15,00	6,00
16,00	20,00	9,00	15,00	8,00
23,00	21,00	13,00	18,00	4,00
18,00	22,00	13,00	17,00	9,00
14,00	15,00	12,00	14,00	6,00
24,00	21,00	15,00	16,00	8,00
18,00	20,00	12,00	20,00	10,00
16,00	15,00	9,00	14,00	7,00
22,00	19,00	14,00	14,00	10,00
16,00	20,00	14,00	17,00	8,00
17,00	14,00	13,00	13,00	9,00
19,00	21,00	11,00	18,00	5,00
12,00	17,00	12,00	13,00	4,00
18,00	19,00	10,00	17,00	5,00
12,00	19,00	9,00	12,00	5,00
22,00	22,00	13,00	17,00	8,00
19,00	19,00	13,00	15,00	8,00
22,00	25,00	15,00	20,00	9,00
21,00	22,00	14,00	20,00	8,00
16,00	20,00	15,00	14,00	10,00
11,00	17,00	15,00	11,00	9,00
13,00	16,00	11,00	9,00	9,00
14,00	15,00	12,00	11,00	7,00
13,00	22,00	15,00	12,00	8,00
19,00	22,00	12,00	19,00	9,00
16,00	17,00	8,00	12,00	8,00
23,00	20,00	15,00	15,00	9,00
21,00	18,00	14,00	16,00	3,00
17,00	22,00	10,00	7,00	8,00
14,00	21,00	12,00	17,00	9,00
15,00	21,00	10,00	16,00	9,00
19,00	14,00	11,00	14,00	6,00
14,00	21,00	13,00	16,00	4,00
20,00	22,00	15,00	18,00	8,00

20,00	23,00	15,00	20,00	8,00
13,00	16,00	9,00	11,00	3,00
15,00	23,00	15,00	17,00	10,00
22,00	21,00	14,00	16,00	9,00
15,00	21,00	12,00	14,00	8,00
14,00	17,00	10,00	12,00	5,00
11,00	17,00	12,00	12,00	10,00
20,00	17,00	14,00	14,00	7,00
18,00	22,00	15,00	16,00	7,00
15,00	13,00	12,00	11,00	4,00
18,00	22,00	12,00	19,00	10,00
14,00	19,00	12,00	17,00	6,00
9,00	11,00	4,00	11,00	8,00
13,00	23,00	13,00	17,00	10,00
21,00	20,00	13,00	13,00	8,00
15,00	13,00	13,00	17,00	7,00
16,00	25,00	14,00	20,00	10,00
20,00	25,00	14,00	16,00	8,00
19,00	24,00	15,00	15,00	7,00
19,00	21,00	12,00	19,00	9,00
11,00	23,00	10,00	12,00	8,00
14,00	17,00	4,00	9,00	5,00
13,00	15,00	8,00	17,00	8,00
18,00	7,00	11,00	10,00	5,00
17,00	15,00	10,00	19,00	10,00
8,00	14,00	12,00	12,00	7,00
13,00	16,00	5,00	12,00	10,00
